# Application of Magnetic Nanoparticles for Reactive Dye Removal from Aqueous Solutions: Practical and Theoretical Approaches

**DOI:** 10.3390/nano16130821

**Published:** 2026-07-02

**Authors:** Iuliana Gabriela Breaban, Imad A. M. Ahmed, Maria Ignat, Loredana Brinza

**Affiliations:** 1 Integrated Center for Environmental Science Studies for the North-East Development Region (CERNESIM), Institute of Interdisciplinary Research/Department of Geography, Faculty of Geography and Geology, Alexandru Ioan Cuza University of Iasi, 11, Carol I Bvd, 700506 Iași, Romania; iulianab@uaic.ro; 2 Department of Earth Sciences, University of Oxford, South Parks Road, Oxford OX1 3AN, UK; imad.ahmed@nanolyse.com; 3 Nanolyse Technologies Ltd., Rutherford Appleton Laboratory, R27 Atlas Centre, Harwell Campus, Didcot OX11 0QX, UK; 4 Faculty of Chemistry, Alexandru Ioan Cuza University of Iași, 11, Carol I Bvd, 700506 Iași, Romania; maria.ignat@uaic.ro

**Keywords:** persistent reactive dye, plain magnetite, adsorption, process kinetic and thermodynamic approaches, adsorbent reuse, wastewater application

## Abstract

This study addresses the critical challenge associated with the removal of reactive yellow dyes from aqueous media and industrial wastewater streams. Owing to their pronounced chemical stability and resistance to conventional degradation techniques, such dyes constitute a substantial environmental concern. In this context, the present work investigates the efficacy of unmodified magnetite nanoparticles (plate-like rounded structures 6–23 nm in size), synthesised under rigorously controlled conditions and well characterised, as high-performance adsorbents for the sequestration of persistent dye species exhibiting limited susceptibility to rapid degradation. The effects of key operational parameters on dye removal efficiency were systematically evaluated to establish optimal treatment conditions. Complete removal of reactive yellow dye (100%) was achieved within 30 min at low initial dye concentrations (20 mg/L) under mildly acidic conditions and continuous agitation. Adsorption equilibrium studies, interpreted using the Langmuir isotherm model, revealed a maximum adsorption capacity of 33 mg/g under optimised conditions. Thermodynamic analysis indicated that the adsorption process is spontaneous (−ΔG° ≈ 46–54 kJ/mol) and endothermic (ΔH° = 21.12 kJ/mol), accompanied by an increase in system disorder (ΔS° = 0.2 kJ/mol × K). Importantly, experiments conducted using real wastewater matrices demonstrated performance comparable to that obtained in deionised water, thereby underscoring the practical applicability of the proposed system. Furthermore, the nanoparticles retained more than 90% removal efficiency after five consecutive adsorption–desorption cycles, employing a basic eluent for dye desorption and surface regeneration. The intrinsic magnetic properties of the adsorbent additionally enable facile recovery and potential reutilisation in secondary applications, including asphalt production. Collectively, these findings highlight the considerable potential of magnetite nanoparticles as effective and reusable adsorbents for wastewater remediation and support further investigation toward pilot-scale implementation.

## 1. Introduction

Large quantities of persistent dyes are employed across various industries to impart colour to products. Examples of such dyes are: triarylmethane (also called triphenylmethane, such as bromocresol green and malachite green), or high-molecular-weight azo reactive dyes such as reactive yellow, reactive red, etc. However, numerous dyes possess toxic properties, potentially exhibiting carcinogenic and mutagenic effects. Dyes introduce toxicity to aquatic life, thereby compromising the aesthetic quality of the environment. These effects extend not only to aquatic biota but also to humans [1]. Dyes typically feature intricate aromatic structures, rendering them stable and challenging to decompose [2,3]. Because of their high solubility, the release of wastewater containing dyes into natural streams and rivers poses a significant issue [4]. Dye concentration in industrially polluted waters varies, generally from 10 to 50 mg/L, with some exceptions where a few hundred mg/L were found [5].

Treating wastewater containing persistent reactive dyes is a real challenge because these dyes exhibit resistance to aerobic digestion and remain stable in the presence of light, heat, and oxidising agents, owing to their complex molecular structure, large molecular size, and resistance to biodegradation [6]. Numerous approaches, encompassing both physical and chemical technologies, have been suggested for efficient dye removal. These approaches include filtration, electrochemical processes [7], coagulation [8], adsorption [9], and catalytic oxidation [10]. Adsorption is an industrial dye treatment technique that is often faster and more efficient than filtration or advanced photocatalytic processes. There is a demand for the development of alternative adsorbents that exhibit both a substantial surface area and minimal diffusion resistance characteristics, besides being environmentally friendly, able to be reused and safe for their final disposal. Iron oxyhydroxide minerals, including ferrihydrite, magnetite, maghemite, goethite, and schwertmannite, are naturally present in various environments such as soils, sediments, and acid mine drainage. These minerals have attracted attention due to their nanoparticulate size and their potential to absorb not only toxic metals [9,11,12,13,14,15,16,17,18], but also organic, persistent and reactive dyes [9,19]. Iron oxyhydroxides that exhibit magnetic properties are highly sought-after as they could be used as nanoparticulate adsorbents that are easily recoverable and potentially reusable in multiple cycles. Before scaling up to industry pilot tests, potential adsorbents undergo rigorous optimisation at the laboratory scale. This optimisation aims to identify the process parameters that maximise the adsorbent’s removal efficiency. Numerous factors influence adsorption performance, including (1) adsorbent characteristics: size and surface properties; (2) adsorbate characteristics: chemistry and concentration; (3) reactor configuration: type and contact time; (4) mixing conditions: type and speed; (5) adsorbent dosage: amount used per unit volume of solution; and (6) solution composition: presence of competing ions, pH, and temperature. Optimising each of these parameters individually in a controlled laboratory environment lays the groundwork for successful process optimisation at larger scales.

As there are tons of dye polluted wastewaters that are discharged untreated to surface waters; hence, there is a continuous interest for researchers in finding new materials to remove dyes (particularly ones with large molecules) from wastewater. Considering individual and/or consecutive and/or simultaneous processes like adsorption, ion exchange and/or degradation, and specifically testing easily available natural nano-minerals (or bio materials or even nano bio composite [20]) that allow repetitive use of the materials, is essential to ensure sustainable byproducts with great performance compared to industrial products that may produce toxic sludge at their discharge. Thus, magnetite, a natural mineral occurring in the environment with double potential to remove organic dyes via adsorption and advanced photodegradation, which also contains magnetic properties that allow easy recovery and reuse, was chosen to be investigated in this study. In addition, magnetite was chosen due to its chemical structure that contain both iron species, Fe(II) and Fe(III), that may play a catalytic role to Fenton like photodegradation processes that might occur, concomitantly or alternatively, and that would led to improvements of dye removal at sites were reactors or treatment plant setup would allow contact with UV daylight—as a photodegradation source [21].

This study investigates the potential of bare, not sophisticatedly engineered magnetite nanoparticles as an easily synthesisable product for removing particularly the reactive yellow dye from water, a very persistent and high-molecular-weight dye that is slowly degradable via photodegradation processes [22]. Thus, new adsorption pathways can be investigated for reactive dye removal from aqueous solution. The novelty of the work consists of using just plain, unmodified magnetite nanoparticles for specifically reactive yellow 84 reactive dye. Its removal was approached via the adsorption process only, with no catalysis or advanced oxidation processes being involved. Laboratory tests were conducted using both simple aqueous solutions and real wastewater samples.

Beyond the standard parameters influencing adsorption (mentioned above), we employed kinetic and thermodynamic modelling to gain deeper insight into the underlying mechanisms. Additionally, the research explored the feasibility of magnetite reuse through multiple adsorption cycles. We investigated optimal eluent solutions that could both desorb the dye and regenerate the adsorbent surface for repeated use.

## 2. Materials and Methods

### 2.1. Chemicals and Databases

Analytical-grade and impurity-free chemicals were used throughout this study. Iron chloride tetrahydrated, FeCl_2_ × 4H_2_O, and iron nitrite nonahydrated, Fe(NO_3_)_3_ × 9H_2_O, salts; ammonium hydroxide, NH_4_OH; and hydrochloric acid, HCl, were purchased from Sigma Aldrich, Dorset, United Kingdom. Reactive Yellow dye has a colour index of RY84 and a chemical formula of C_50_H_24_C_l2_N_14_Na_10_O_30_S_10_ that has a solubility in water at 293 K of 70 g/L.

**Figure 1:**
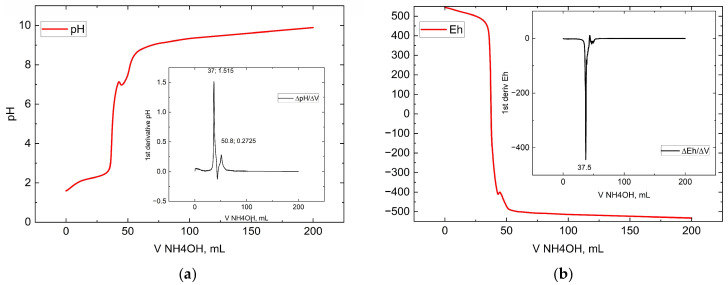

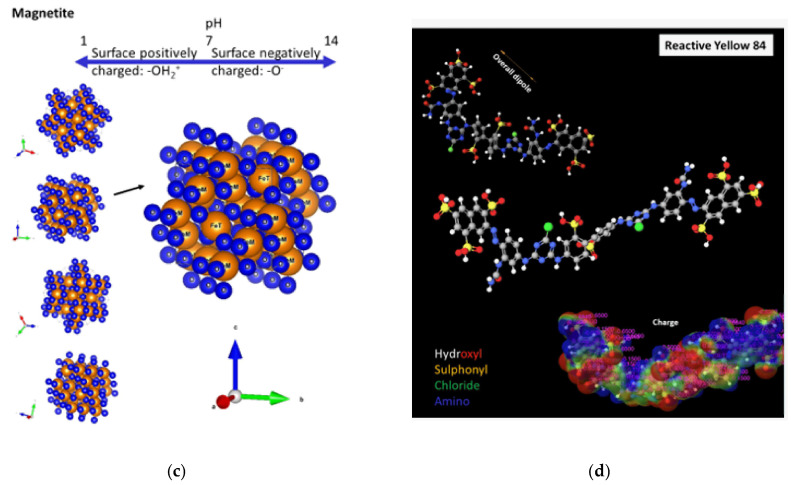
pH (**a**) and redox potential (**b**) profiles during magnetite synthesis as a function of base volume added during the synthesis. (**c**) Structural representation of magnetite (tetrahedrally (Fe-T) and octahedrally (Fe-M) coordinated iron atoms in orange and oxygen atoms in blue) from different angles with surface charge properties described from point of zero charged results and (**d**) structural representation of Reactive Yellow 84 dye with colour coded structural and functional groups explained, and which are available to bind at adsorbent surface.

The displays of magnetite and reactive yellow molecule structures, as shown in Figure 1, were carried out with the VESTA v. 3.4.0 [23] and MolView v 2.4 (2024) [24] software, respectively using run COD ID: 9006189.cif file from the Crystallography Open Database [25] for magnetite and SID: 93942-66-6 from the PubChem database [26] for RY 84.

### 2.2. Synthesis of Magnetic Nanoparticles

The magnetic nanoparticle synthesis approach and related technical know-how used in this work were contributed by Nanolyse Technologies Ltd. as part of the collaborative experimental work reported in this manuscript. Magnetite synthesis was carried out in a 600 mL Teflon-made closed batch reactor by precipitation method. FeCl_2_ × 4H_2_O and Fe(NO_3_)_3_ × 9H_2_O salts (Sigma Aldrich, Dorset, United Kingdom) were used in 1:1 mixtures to ensure the initial Fe(II)/Fe(III) mole fraction of 0.5. The initial pH was 1.579, and it was raised to 10 using 5 M ammonium hydroxide by adding very fine and precise microvolumes using a high-resolution potentiometric titrator (ManThech, East Sussex, United Kingdom). This high-resolution potentiometric titrator was used to sensibly dose the reactants in microvolumes and at optimum time intervals and under controlled flow rates (initially pretested) that ensured homogeneity of the system and avoided hot reaction points during nanoparticle formation. Magnetite nanoparticles were synthesised under strictly controlled conditions. An O_2_-free environment was maintained by purging the reactor with N_2_ gas during synthesis. Additionally, pH and redox potential (Eh) were continuously monitored.

At the end of the synthesis, the slurry was carefully transferred to an oxygen-free vacuum oven equipped with a blowing N_2_ gas line and washed with deoxygenated deionised water (18 mΩ) several times until Total Dissolved Solids was lower than 30 ppm (equivalent to 45 μS cm^−1^ in electrical conductivity). The washed slurry was dried under vacuum overnight and the resulting solid was ground to a fine powder, well-sealed and stored until it was used for the adsorption tests.

### 2.3. Characterisation of Magnetic Nanoparticles

HITACHI—HT7700 transmission electron microscope (TEM) was used to investigate particle size and morphology as well as to collect selected area electron diffraction (SAED) spectra. Images were recorded at different magnifications up to 100 k, at a voltage set to 10 eV.

To determine particle size, we employed Small-Angle X-Ray Scattering (SAXS) at the Diamond Light Source (UK) using a XEUSS 3.0 instrument, France on the LabSAXS beamline. This powerful device boasts two X-ray sources: a high-intensity Excillium Gallium MetalJet (9.2 keV) and a Molybdenum Microsource (17.4 keV), of which the Molybdenum source was used in this research. Data was collected using an Eiger R 1M detector (DECTRIS Ltd. Baden-Daettwil, Switzerland) with a pixel size of 75 μm positioned 1.5 m from the sample. The X-ray beam was 200 μm in diameter, and the entire measurement process was conducted in air. This setup allows capturing particles or pores in sizes up to ca. 40 nm. SAXS profile measured in reciprocal space and Fourier-transform SAXS data were processed to obtain a distribution of distances between scattering pairs in the sample, namely the P(r) function in real space.

To identify the mineral phases, present in the sample, we employed X-ray diffraction (XRD) using a Shimadzu diffractometer equipped with a Cu Kα radiation source. The instrument settings for data collection were: 40 kV, 30 mA, scanning range of 10 to 90 degrees (2θ), scan speed of 2 degrees per minute, and a step size of 0.02 degrees. The obtained diffraction patterns were then analysed using the MATCH! 4. software (Crystal Impact GbR, Bonn, Germany) to identify the present mineral phases. Additionally, confirmation of magnetite presence was achieved by consulting several databases such as the Crystallography Open Database, RRUFF, and MinDat.

Fourier-Transform Infra-Red (FTIR) spectroscopic measurements were carried out at Diamond Light Source using a Nicolet (from Thermo Fisher Scientific, Loughborough, United Kingdom) device coupled to an ATR device equipped with a diamond crystal. FTIR spectra were in attenuated transmission reflectance from 4000 to 600 cm^−1^, at a resolution of 2 cm^−1^. Each spectrum is a mean of 512 scans collected at a resolution of 32.

Raman spectroscopic characterisation was carried out at the Diamond Light Source, United Kingdom, using a Renishaw device that uses a 473 nm laser beam and was coupled to an optical microscope. A total of 512 spectra were averaged at a resolution of 32 within an interval of 100–3200 cm^−1^.

X-Ray Absorption Spectroscopy, XAS, specifically X-Ray Absorption Near-Edge Spectroscopy, XANES, measurements were carried out at the I18 beamline of the Diamond Light Source synchrotron, UK, to gain information about the iron oxidation state in the bulk material. Fe XANES spectra were collected in fluorescence mode at an energy interval of 100 eV below to 300 eV above the Fe k edge (7112 eV). XANES spectra of representative Fe(II) and Fe(III) standards were collected for FeO and Fe_2_O_3_ respectively. Data normalisation and background subtraction were carried out in Athena from Demeter with Strawberry Perl software, United States [27]. Linear combination fitting of XANES and pre-edge features allowed semi-quantification of Fe(II) and Fe(III) species.

The Quantachrome NOVA 2200E BET Surface Area Analyzer (Quantachrome Corporation, Boynton Beach, FL, USA) was used to determine nanoparticle surface area. The measurements were carried out at −196 °C (in liquid nitrogen) after the samples were prepared by outgassing under a high vacuum at room temperature (according to IUPAC and ISO 9277 [28]). The Brunauer–Emmett–Teller (BET) model was used to calculate specific surface area. Pore volume was estimated directly from the nitrogen adsorption isotherm at a relative pressure of P/P0 = 0.95.

### 2.4. Adsorption Experiments

Adsorption experiments were carried out using 100 mL beakers as batch reactors. Each reactor contained 50 mL of dye solution at varying pH and dye concentrations. A small amount (0.05 g) of dried magnetite nanoparticles was added to each reactor, and the mixture was stirred for approximately 2 h on a magnetic stirrer [29]. To monitor dye removal over time, small samples (around 3 mL) were periodically withdrawn from the reactor at 10, 20, 30, 60 and 120 min. These samples were then filtered through a 0.2 µm membrane filter to remove any remaining nanoparticles. Finally, the filtered solutions were analysed using a UV-VIS spectrophotometer set to a wavelength of 400 nm to determine the concentration of dye remaining in the solution. No interference was found to affect the measurements, as the dye is very stable, also as a consequence of its persistence and refractoriness. This method has a limit of quantification of 0.02 mg/L.

All the experiments were run in duplicate, including controls, and the results were presented as kinetic profiles of adsorption capacity (q, mg/g) and removal efficiency (E, %).

The amount of dye adsorption per g adsorbent was calculated using the expression of uptake capacity as described in Equation (1).

(1) $$q=\frac{\left(C_{i}-C_{t}\right)\cdot V}{m}$$

where q, in mg/g, is the mass of dye adsorbed per gram of dried weight magnetite; Ci and Ct are the initial concentration and concentration at time t of the dye in mg/L; V is the volume of solution and m is the mass of the magnetite, expressed in g.

Dye removal efficiency, E (expressed in %), is defined as

(2) $$E=\frac{\left(C_{i}-C_{t}\right)\cdot 100}{C_{i}}$$

Below, particular setups for each experimental set of investigation (the effect of pH, dye concentration, particle concentration, temperature, mixing effect and applicability to real wastewater) are briefly described.

*The Effects of Key Parameters for Magnetite Dye Adsorption*

To assess the impact of key parameters on real-world applicability, we conducted lab-scale adsorption experiments investigating the following factors:

*(1) pH Effect:* The initial solution pH was varied from 4 to 9 to encompass a broad range of environmental conditions. A magnetic stirrer ensured mixing at 200 rpm, and the experiments were run at room temperature with a fixed initial dye concentration at approx. 20 mg/L. A pH-stat titrator (SI Analytics, White Plains, NY, United States) maintained a constant pH throughout the adsorption process.

*(2) Initial Dye Concentration:* The initial dye concentration was varied from 5 mg/L to 100 mg/L while maintaining a pH of 4.2 and mixing at room temperature. This range was chosen to cover dye concentrations above discharge limits as well as concentrations as high as ecotoxicological limits (LC50 ≥ 100 mg/L).

*(3) Particle Concentration:* The effect of adsorbent dosage was investigated by varying the amount of nanoparticles used (0.6 g/L to 2 g/L) in batch reactors. The experiments were conducted at room temperature at a pH of 4.2. The range was chosen to obtain information about the adsorbent saturation limit and also offer guidance on the optimal dosage needed to be used for a certain pollutant concentration range.

*(4) Temperature Effect:* We investigated the impact of temperature on adsorption using a well-mixed 25 mL batch system. The experiments were conducted at 20 °C, 30 °C, 40 °C, and 50 °C. Initial dye concentrations of 20 mg/L, 50 mg/L, and 100 mg/L were tested alongside a fixed adsorbent concentration of 1 g/L. During the adsorption process, 3 mL supernatant aliquots were withdrawn at intervals of 10, 20, 30, 60, and 120 min. These samples were filtered through cellulose nitrate membranes and analysed using UV-VIS to determine dye concentration.

*(5) Mixing Effect:* The influence of mixing type and dynamic regime on removal efficiency was explored by comparing the static regime with various dynamic setups. Three dynamic scenarios were evaluated using batch reactors: vibrational mixing at 30 vibrations per minute; magnetic mixing at 200 rpm and 300 rpm. All the experiments used identical conditions: pH 4, temperature 20 °C, initial dye concentration 20 mg/L, and adsorbent dosage 1 g/L.

*(6) Real-World Wastewater Applicability:* To evaluate the impact of a complex wastewater matrix on dye removal efficiency, an adsorption study was conducted using surface water (The Blue Lake, Baia Sprie, Maramures, Romania) featuring physicochemical characteristics as referenced in Maftei et al., 2025 [30], that was spiked with reactive yellow dye. Blue Lake water was spiked to reach an initial dye concentration of approximately 20 mg/L. The experiment mimicked optimal conditions identified previously (200 rpm stirring, room temperature, and pH 4.2) and used the same adsorbent dosage (1 g/L). Similar to the previous experiments, 3 mL samples were withdrawn at predetermined intervals, filtered using 0.22 µm membrane filters, and analysed by UV-VIS to determine dye concentration. Removal efficiency in wastewater was then compared to the results obtained with deionised water.

Kinetic profiles were expressed as removal efficiency (E) and adsorption capacity (q). The q data was further analysed using various common kinetic models, which are described in detail below.

### 2.5. Adsorption Kinetic Models

Adsorption modelling was carried out to extract empirical information about the mechanism of reactive yellow removal by magnetite. It also allowed us to calculate weighted values for uptake capacities at equilibrium.

Pseudo-first-order (PFO), pseudo-second-order (PSO), Elovich, and Weber–Morris intraparticle diffusion models [31,32,33,34,35,36,37,38,39,40,41] were used to model the kinetics of dye adsorption. Detailed information about the kinetic models used in this study is presented in the Supplementary Information.

### 2.6. Adsorption Isotherms

The set of experiments designed to obtain adsorption isotherms was conducted in batch reactors at pH 5 and room temperature (approx. 25°C) by varying adsorbate concentrations (0–100 mg/L) at a fixed adsorbent concentration (1 g/L). The data were plotted as uptake capacities at equilibrium versus adsorbate concentration in solution at equilibrium. The modelling was performed by using the Langmuir [42,43,44], Freundlich [1,45], and SIPS isothermal models, commonly used to derive empirical information about adsorption mechanisms. Further details about adsorption isotherm models [46] as well as the Langmuir separation factor, R_L_, are presented in the Supplementary Information. Origin software (2023, academic version 10.0.0.154) [47] was used to perform nonlinear fitting of the kinetic and isotherm modelling. Statistical parameters were calculated for each model, and statistical models such as ANOVA and *t*-test were included. We evaluated how well the model fit the experimental data using several metrics. These included minimising the chi-square value (ideally close to zero) and maximising the coefficient of determination (R^2^) and adjusted R^2^ (both aiming to be as close to one as possible).

### 2.7. Adsorption Thermodynamics

The adsorption process thermodynamics were evaluated by calculating key parameters like Gibbs free energy (ΔG), enthalpy (ΔH), and entropy (ΔS) changes. This calculation employed the Langmuir constant (K_L_, L/mg) to derive the equilibrium constant (Kc, Kc = K_L_ × MW × 55.5 × 1000). The approach assumes a constant Gibbs free energy for all the adsorption sites.

Gibbs free energy change was calculated as follows:

ΔGº = −RT ln Kc(3)

where R is the gas constant (8.314 J/mol x K), T is temperature in Kelvin, and Kc is the equilibrium constant (dimensionless). The equilibrium constant was derived from the Langmuir constant, K_L_, expressed in L/mg, which was converted to a dimensionless constant following an approach suggested by Tran et al. (2017) [48].

The K_L_ units conversion to Kc was considered using approaches originally developed by Zhou and Zhou (2014) [49], and Milonjic (2007) [50], where enthalpy (ΔHº) and entropy (ΔSº) changes are calculated from the slope and intercept of the plot (ln Kc vs. 1/T) of the linearised form of the Van ‘t Hoff equation as described in Equation (4).

(4) $$\ln K_{C}=\frac{-∆H{}^{\circ}}{R}\;\frac{1}{T}+\frac{∆S{}^{\circ}}{R}$$

To avoid potential experimental data fluctuations/inconsistencies from handling/evaporations errors or slight desorption that might have occurred at the end of adsorption at high temperatures, weighted values of q_e_ and Ce were chosen for drawing the temperature-related adsorption isotherms by fitting the experimental data to the PFO kinetic model and to Expo decay polynomial, respectively.

### 2.8. Reuse of Adsorbent

Magnetite reuse tests were carried out in batch reactors as consecutive adsorption–desorption cycles. As eluents, for the dye recovery by desorption and surface conditioning and potential functioning, HCl 0.1 mM, H_2_O and NaOH 0.1 mM solutions were chosen to cover a whole range of pH: 4—acidic solution, 7—H_2_O, and 9—basic solution respectively. For this set of experiments, the process conditions chosen were: adsorption—C_dye_ = 20 mg/L, C_ads_ = 1.0 g/L, pH 4.2, T = 20 °C; dynamic regime—magnetic stirring at 200 rpm, contact time 60 min; desorption—C_eluent_ = 0.1 mM HCl; 0.1 mM NaOH and DI H_2_O brought to pH 7 with NaOH 1 mM, pH 4, 7 and 10, respectively, C_ads_ = 1.0 g/L, T = 20 °C; dynamic regime—magnetic stirring at 200 rpm, contact time 10 min. To avoid adsorbent loss between cycles, the systems were left to settle; hence, the nanoparticles were fully recovered onto a magnetic bar before filtration ca. 5 min before supernatant collection, filtration and analyses for dye concentration. The desorbed dye percentage was calculated as a percent of the adsorbed amount in the previous cycle.

## 3. Results and Discussion

### 3.1. Nanoparticle Synthesis

The profiles of pH and Eh as a function of the volume of base added to nanoparticle synthesis, as monitored by potentiometric titration, are displayed in Figure 1.

The pH profile shows a rapid increase after approx. 37 mL of base added, followed by another lower slope at ca 50 mL of base being added. The first derivative of the pH/V led to the calculation of two equivalent points at pH values of 4.27 and 7.69. These endpoints may coincide with the completion of reacting Fe(II) and Fe(III) from the system during magnetite formation.

Figure 1b displays the profile of Eh and its first derivative (inset), recorded during magnetite formation. The Eh profile shows a deep slope of the potential after ca 37.5 mL of 5 M NH_4_OH is added to the system, which coincides with the first equivalent point depicted from the pH titration profile.

Figure 1c displays the structural representation of magnetite nanoparticles, as produced by VESTA v. 3.4.0 [23], showing tetrahedrally (FeT) and octahedrally (FeM) coordinated Fe(II) and Fe(III) atoms (orange) interlayered with O atoms (blue) that can act as surface functional groups as a function of environmental pH. Additionally, it includes info about surface charge properties as a function of pH.

MolView v 2.4 (2024) [24] drawing of reactive yellow large molecule is displayed in Figure 1d and shows functional groups available to potentially bind at magnetite surface as well as electrons charge density and overall dipole vector. 

### 3.2. Characterisation of Magnetic Nanoparticles

**Figure 2:**
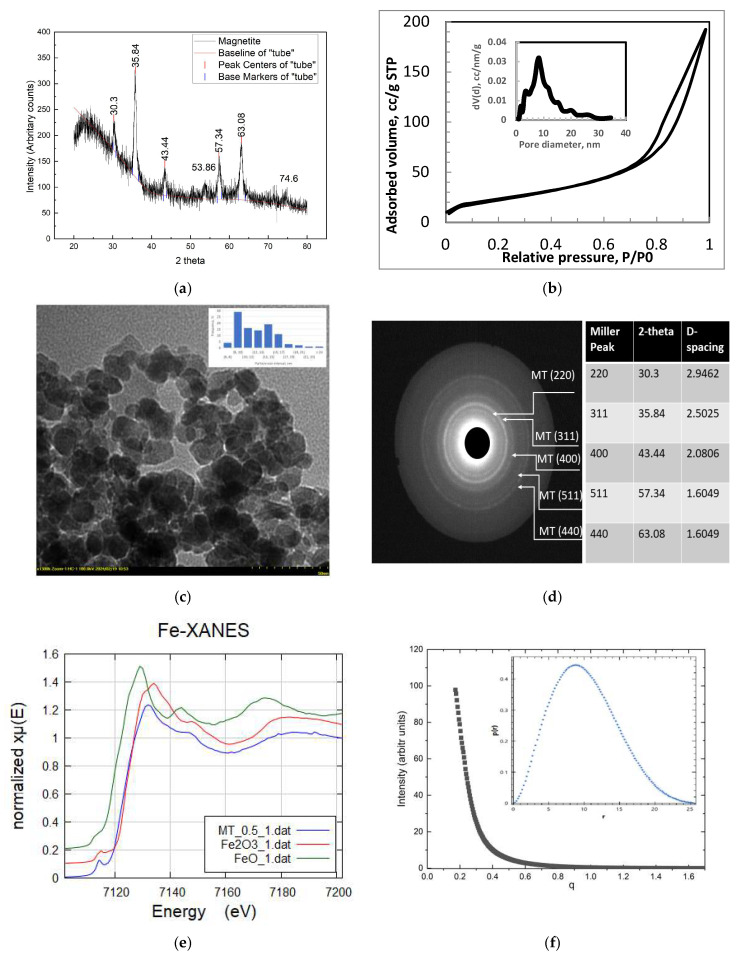
X-ray diffraction (**a**); BET isotherm (**b**); TEM micrograph (**c**); selected area electron diffraction, SAED (**d**); Fe-k edge X-ray absorption near edge spectroscopy (XANES) spectra of magnetite nanoparticles along with two representative Fe(II) and Fe(III) standards, FeO and Fe_2_O_3_, respectively (**e**) and Small-Angle X-ray Scattering (SAXS) profile and calculated P(r) function (inset) (**f**) characterisation of magnetic nanoparticles used in the current study.

*X-ray Diffraction:* XRD diffractogram of the magnetite used in this study is displayed in Figure 2a. The XRD pattern shows high intensity diffraction peaks at 30.3, 35.8, 43.4, 53.7, 57.3, 63.08, 74.6 2-theta degree that match cubic magnetite (Space Group: Fd3m; Cell Parameters: a = 8.396 Å) of Miller indexes: (220); (311); (400); (422); (511); (440) and (533), respectively [51,52]. After using the Scherrer method and Williamson–Hall plot to estimate the crystallite size, the results showed that the average particle sizes are 17 nm and 53.32 nm, respectively.

*Electron Microscopy:* The TEM micrograph of magnetite nanoparticles exhibits crystalline, rounded particles and plate-like structures with an average size of 12.23 nm (Figure 2c). The histogram (inset in Figure 2c) shows a narrow particle size distribution of 6-23 nm. The selected area electron diffraction (Figure 2d) displays six concentric circles at d-spacings that correspond to the indexed Miller peaks of the X-ray diffraction pattern (Figure 2a). These results confirm the mineralogy of the magnetite nanoparticles. Comparing the TEM average size (12.23 nm) from a 6–23 nm range and the Scherrer method results (17 nm) with the Williamson–Hall plot results, it can be observed that the WH plot gave a higher value (53.32 nm), which can be explained by the peak-broadening effects of strain being removed.

*BET Measurements:* The BET adsorption raw diagram is displayed in Figure 2b and features a type IV isotherm, according to IUPAC classification, that is characteristic of mesoporous materials [53]. The presence of mesopores indicates the existence of empty spaces between agglomerated nanoparticles. The isotherm is accompanied by an H4 hysteresis loop, indicating that pores are narrow and of slit shapes, given by particles of nonregular shapes [54]. The fitted results show that nanoparticles have a high surface area of 84.978 m^2^/g (R^2^ = 0.9994). The calculated pore volume and pore diameter were of average values of 0.945 ± 0.247 cc/g and 1.12 nm, respectively. The mean of the pore size diameter has been calculated from Density Functional Theory (DFT) considering the slit pore QSDFT equilibrium model that led to a broad distribution with an average of approx. 8 nm [54]. The role of magnetite surface area in dye adsorption is mainly related to uptake performance. Generally, the greater the surface are the better the dye uptake performance.

FTIR and Raman analyses confirmed a typical surface structure of magnetite with characteristic Fe-O vibrations and adsorbed water (See Supplementary Information Figure S1a,b).

*FTIR Analysis:* The FTIR spectrum collected for the magnetic ferrite used in this study is displayed in Figure S1a. The most prominent signal in the region of wavenumbers 1621–1630 cm^−1^ is assigned to the deformed symmetric bending mode of OH of adsorbed water molecules at the magnetite surface. The absorption bands at wavenumbers 1321 and 1067 cm^−1^ correspond to the stretching vibration of the primary –OH group and the secondary –OH group, respectively. There is also a well-known vibration at wavenumber 536 cm^−1^, known for typical magnetite and assigned to Fe-O vibrations [2,3]. FTIR investigation of hydrothermally synthesised magnetite by Saranya et al. (2015) showed two absorption bands at wavenumbers 584 cm^−1^ and 442.03 cm^−1^, which correspond to the vibration of tetrahedral and octahedral Fe-coordinated sites, respectively [55]. According to the above and the fact that the divalent Fe(II) ions are in tetrahedral coordination, whereas the Fe(III) ions are in octahedral coordination, the peak observed from wavenumbers 536–560 cm^−1^ may be assigned to an average of the two different vibrations. In our magnetite synthesis method, the initial concentration of Fe(III) was 0.1 M and of Fe(II) was 0.05 M.

*Raman Analysis:* In Figure S1b, the Raman spectrum of the vacuum-dried magnetic ferrite used in the current study is shown. It consists of spectra that has strong signals at 220.5 cm^−1^, 286.9 cm^−1^, 793.6 cm^−1^, 914.6 cm^−1^, 1084 cm^−1^, 1556.8 cm^−1^, 2329.9 cm^−1^, and broad signals at ca 503.9 cm^−1^, 1358.1 cm^−1^. Most of the Raman spectra found in the literature are collected using laser beams of 514 nm, 532 nm, 633 nm and 785 nm [55,56,57,58,59,60,61,62]. In all the studies, the spectra look slightly different. Thus, no exact comparison could be made. Still, a general description of common Raman vibration bands for iron oxyhydroxides was compiled by Hanesch (2009) [59] from Cornell and Schwertmann’s (2003) [63] work, who showed that for magnetite common bands are located at 670 cm^−1^, followed by ones at 540 cm^−1^ and 310 cm^−1^. The common bands were found for reference material used as a standard in a paleomagnetic laboratory [59]. Additionally, according to Shebanova et al.’s (2003) work, magnetite is suitable for oxidation under a laser beam, depending on beam intensity [60,64].

There are no magnetite spectra in the literature collected with a laser at a lambda of 473 nm. However, a Raman study that collected magnetite spectra with a laser close to 473 nm, exactly 488 nm, is investigating the oxidation of magnetite nanoparticles [65]. They suggested that with a limited penetration depth of a 488 nm laser, the oxidation of Fe(II) from the magnetite surface can be assessed by the Raman mode (A1g) near 660 cm^−1^. For maghemite, this mode is split into two equal components around 660 cm^−1^ and 710 cm^−1^. As magnetite transforms to maghemite via oxidation, this split is more obvious. In addition, they suggest a lattice distortion as oxidation occurs and progresses. They support this distortion by shifts in the 2 bands around 300 cm^−1^ and 530 cm^−1^ corresponding to T2g modes of magnetite changing with oxidation [65]. Thus, among all the vibrations recorded in the current spectrum, the one at 286 cm^−1^ could probably be assigned to magnetite. The interpretation of the rest of the bands remains open for further studies/investigations and readers’ opinions.

*X-Ray Absorption Spectroscopy (XAS):* It is generally known that the two types of Fe ions in magnetite are coordinated as tetrahedral (Fe(II)) sites and octahedral (Fe(III)) sites, and that the larger octahedral sites are occupied by twice as many octahedral sites as tetrahedral sites.

The XANES spectra of the magnetite used in the current study, displayed in Figure 2e among two other spectra of FeO and Fe_2_O_3_, chosen as representative Fe(II) and Fe(III) standards, show that magnetite spectra feature a pre-edge at 7114.5 eV and the adsorption edge at 7122.35 eV. The pre-edge feature is correlated to the Fe oxidation states found in the structure. The FeO standard’s binding energy near 7112.71 eV indicates dominance of Fe(II) within the structure.

Conversely, the Fe_2_O_3_ standard’s value closer to 7115.7 eV suggests a prevalence of Fe(III) in the nanoparticles. Assessment of the pre-edge position of the magnetite spectra suggests that Fe is found within magnetite as Fe in both Fe oxidation states. To determine the percentages of Fe species in the magnetite structure, linear combination fittings of various regions of XANES spectra were carried out (see Supplementary Information Figure S2a). Linear combination fittings of the magnetite spectra within the −20 eV before the absorption edge and 150 eV post absorption edge interval, using the two selected standards as representative Fe(II) and Fe(III) spectra, showed that 72.2% Fe is similar to Fe(III) and 27.8% Fe is similar to Fe(II) within the magnetite structure. Further evidence of these semiquantitative results (similar Fe(II) and Fe(III) percentages) was obtained by LCF of other XANES regions (see Supplementary Information Figure S2b,c).

Post edge features that can be observed are: the maximum of the white line at 7132.39 eV, a shoulder at 7147.13 eV followed by consecutive scattering signals with minimum amplitude at 7161.54 eV and max 7184.77 eV and further small scattering signals at approximately 7212 eV, 7226 eV and 7249 eV. These features are averaged scattering signals of Fe neighbouring atoms.

*SAXS Measurements*: SAXS raw data expressed in scattering vector, q, (1/nm) are presented in Figure 2f, and the calculated particle radius is shown as an insert in Figure 2f. SAXS Measurements: SAXS profile measured in reciprocal space and Fourier-transform SAXS data to get a distribution of distances between scattering pairs in the sample, namely P(r) function in real space (inset) shown in Figure 2f, reveal aggregation and/or polydispersity effects coming from the observed upturn of the SAXS profile at low q values. The calculated power-law exponent (α = 3.7812) indicates the structure of magnetite scatterings is more like surface fractals [66], suggesting a rough surface of the synthesised particles. The determined P(r) function (Figure 2f, inset) shows the magnetite nanoparticles have a maximum size of 25 nm, the results being in good agreement with the TEM-derived particle size distribution.

### 3.3. Adsorption Experiments

**Figure 3:**
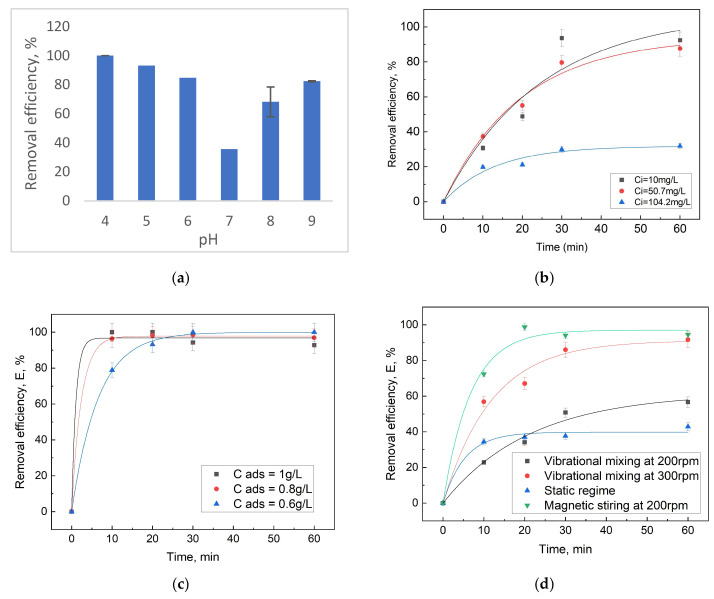
pH profile of reactive yellow removal efficiency by magnetite nanoparticles under the following process conditions: pH = 4–9, 120 min reaction time, C_dye_ approx. 20 mg/L, C_ads_ = 1 g/L, T = 25 °C; dynamic regime: magnetic stirring at 200 rpm (**a**); kinetic profile of dye removal efficiency at various process paraments investigated: (**b**) dye concentrations at following process conditions: pH 4.2, T = 25 °C, C_ads_ = 1 g/L, C_dye_ = 10–104 mg/L, mixing regime: dynamic, magnetic stirring at 200 rpm; (**c**) adsorbent concentrations (dosage) at following process conditions: pH 4.2, T = 25 °C, C_dye_ = 20.4 mg/L, C_ads_ = 0.6–1 g/L, mixing regime: dynamic, magnetic stirring at 200 rpm; and (**d**) under static vs. dynamic regimes, using magnetic vs. vibrational mixing at various speed (200 rpm vs. 300 rpm) at following process conditions: pH 4.2, T = 25 °C, C_dye_ = 20.4 mg/L, C_ads_ = 1 g/L, mixing regimes: static vs. dynamic, dynamic: magnetic stirring at 200 rpm vs. vibrational at 200 rpm and vibrational mixing at 200 rpm vs. 300 rpm.

This experiment examined how well magnetic nanoparticles remove a persistent yellow dye (reactive yellow) from water at different pH levels. The results are in Figure 3a. They show that the nanoparticles were very effective at removing the dye in acidic and mildly acidic and alkaline environments (pH 4–5 and 8–9). Nearly all (90–100%) of the approx. 20 mg/L dye was removed at pH 4 and 5. However, at neutral pH (7), dye removal was significantly slower, with only 35% removed. Interestingly, under mild alkaline conditions, the efficiency increased to 68% at pH 8 and 82% at pH 9. These findings suggest that magnetic nanoparticles are promising for removing this dye from water, especially in acidic and mildly acidic and mild alkaline conditions, but not neutral. If the Point of Zero Charge, PZC, of magnetite is considered (at pH of 6.5–7 [67]), an adsorption mechanism might be suggested as follows: under acidic conditions, the reactive yellow binds to the magnetite surface via anionic functional groups (i.e., sulphate) of its structure at positively charged surface sites of magnetite. As pH increases to near PZC (where the magnetite surface is zero charged or the number of positively charged sites equals the negatively charged sites, at about pH 6.5 [68]), the adsorption decreases.

The efficiencies of dye removal at varying dye concentrations, as presented in Figure 3b, show that a maximum removal efficiency of 90% to 100% of dye was reached after 30 min at an initial dye concentration of 10 mg/L. As the initial dye concentration increases, the dye removal efficiency slightly decreases to 80%—for an initial dye concentration of 50.7 mg/L and to only 30%—for an initial dye concentration of 104.2 mg/L. This trend suggests that absorbent surface sites tend to saturate as the initial concentration of dye increases in the aqueous solution.

Further to the above outputs, the effect of particle concentration/adsorbent dosage was considered to remove 20.4 mg/L persistent reactive dye from aqueous solutions using 0.6 to 1 g/L of nanoparticles (Figure 3c) at an optimum pH of 4.2. The results showed that 80% of the dye was removed within the first 10 min when the lower adsorbent dosage of 0.6 g/L was used, whereas at increasing dosage to 0.8 and 1 g/L, all 20 mg/L dye was removed very fast, within 10 min. This trend indicates that increasing adsorbent dosage not only increases the removal efficiency but also leads to faster pollutant removal. These results suggest using more adsorbent to effectively remove the pollutant and meet environmental regulations for wastewater with high reactive yellow dye concentrations. However, there is a practical limit to how much adsorbent can be used.

The impact of mixing regime and type on dye removal efficiency was investigated. A static system (no mixing) was compared to dynamic systems with two types of mixing: magnetic stirring and vibrational mixing at 200 rpm and 300 rpm (Figure 3d). All the experiments used an adsorbent dose of 1 g/L at the optimal pH and an initial dye concentration of approximately 20.4 mg/L (reactive yellow). In the static system, the adsorbent removed only 43% of the dye within 10 min, reaching apparent equilibrium. Magnetic stirring at 200 rpm resulted in slower dye removal, achieving a maximum efficiency of 50% after 2 h. However, increasing the vibrational mixing speed to 300 rpm significantly enhanced dye removal efficiency to 92%, reaching equilibrium within 1 h. This suggests that faster mixing promotes mass transfer of the dye molecules to the adsorbent surface. Interestingly, magnetic stirring at 200 rpm achieved complete dye removal within 20 min, indicating superior efficiency compared to vibrational mixing at the same speed.

These results suggest that magnetic stirring is the most effective mixing method for reactive yellow removal by magnetite nanoparticles under these experimental conditions. It is important to note that the existing literature primarily focuses on the influence of mixing speed and time on dye adsorption using different materials. This study highlights the additional factor of mixing type, which can significantly impact the efficiency of the adsorption process [69,70,71,72,73,74].

Previous studies have explored the impact of mixing speed on dye removal efficiency, with the reported effects ranging from negligible influence [70] to increased efficiency with higher speeds [69,75]. The typical mixing speed range investigated is up to 500 rpm, with 100–200 rpm being the most common [71]. However, the existing literature lacks studies directly comparing static vs. dynamic mixing, or magnetic vs. vibrational mixing, in the context of dye adsorption.

Tangentially relevant are investigations comparing batch reactor vs. column reactor configurations for dye removal efficiency. For example, Akar et al. (2021) reported superior decolourisation of Basic Blue B7 using an eco-friendly sorbent in a batch system (96%) compared to a continuous system (56%) [74]. Interestingly, their modelling suggested that for certain systems, very high mixing speeds (e.g., 260 rpm in their study) could lead to decreased removal efficiency. This behaviour may be attributed to the characteristics of the specific adsorbate-adsorbent system, potentially causing the desorption of weakly bound dye molecules due to increased Brownian motion [74].

The influence of temperature on dye removal efficiency is depicted in Figure S3a,c,e. In all the cases, increasing temperature resulted in a decrease in removal efficiency. At an initial dye concentration of 20 mg/L, efficiency dropped from 100% at 20 °C to 84% at 50 °C (approximately a 20% decrease). Similar trends were observed for initial dye concentrations of 50 mg/L (64% at 20 °C to 16% at 50 °C) and 100 mg/L (35% at 20 °C to 12% at 50 °C).

**Figure 4:**
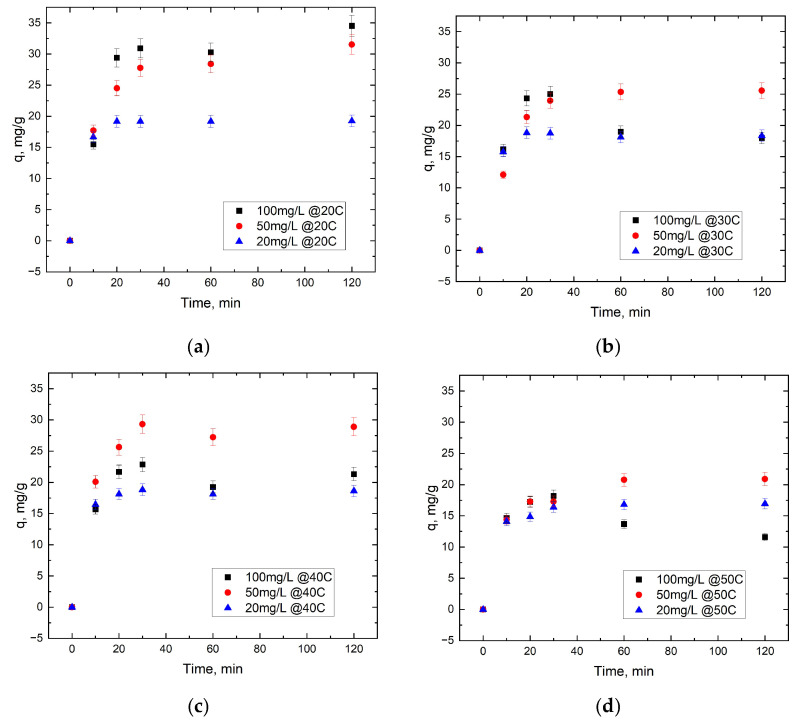
Kinetic profiles of experimental results of magnetite uptake capacity for reactive yellow at various initial dye concentrations and temperatures of (**a**) 20 °C, (**b**) 30 °C, (**c**) 40 °C and (**d**) 50 °C. Process conditions: C_i dye_ = 20–100 mg/L. C_ads_ = 1 g/L, pH 4.2, under dynamic regime with magnetic mixing speed of 200 rpm.

Examining the relationship between adsorption capacity (q) and time across various temperatures reveals interesting trends (Figure S3b and Figure 4). At 20 °C, q increases with higher initial dye concentrations for the first 30 min, indicating efficient uptake. However, this trend shifts at higher temperatures (30 °C and above). Here, q starts to decrease after 60 min for higher dye concentrations, suggesting potential desorption of previously adsorbed dye molecules. This effect becomes even more pronounced at even higher temperatures (40 °C and above), implying that thermal factors, possibly increased Brownian motion of the dye molecules and particles in solution, hinder adsorption performance at these combined extremes.

Further mechanistic insights can be gleaned from a combined analysis of q (adsorbent capacity) and E (a wastewater cleanness parameter) profiles, particularly with respect to the time required to reach equilibrium (Figure S3). As the initial dye concentration increases in these experiments, the observed trends suggest an optimal range for capturing mechanistic information.

At low initial dye concentrations (20 mg/L), equilibrium is reached within 20–30 min. However, the near-complete dye removal (E ≈ 100%) observed here indicates high efficiency but not necessarily surface saturation of the adsorbent. This conclusion is supported by the minimal amount of dye remaining in the solution, as it was in the adsorption supernatant. Conversely, at a medium initial concentration (50 mg/L), both equilibrium and a plateau in q (within 30 min) suggest that saturation is being approached. Efficiency values here range from 40 to 50%, indicating some remaining dye in solution and thus the adsorbent being close to saturation. Finally, at the highest initial concentration (100 mg/L), the low removal efficiency (15–30%) combined with a decrease in q and E after 30 min strongly suggests oversaturation. The limited number of available binding sites on the adsorbent restricts further dye removal at this high concentration. Additionally, the observed desorption at high concentrations and temperatures further supports the idea that the adsorbent surface has reached its saturation limit.

Nanoparticle stability tests were carried out by X-ray diffraction, scanning the samples before and after adsorption (at pH 4). The results presented in Figure S1c show that Miller peaks in magnetite post adsorption are similar to initial non-reacted magnetite nanoparticles. A minor shift in all the XRD peaks towards lower theta (by approx. 0.22 theta degree) may indicate slightly increased unit cell post adsorption, which might be due to the surface coverage post adsorption. However, the results indicate that magnetite nanoparticles remain stable post adsorption under optimum pH conditions with no new mineralogical phase being observed.

### 3.4. Adsorption Kinetics

**Figure 5:**
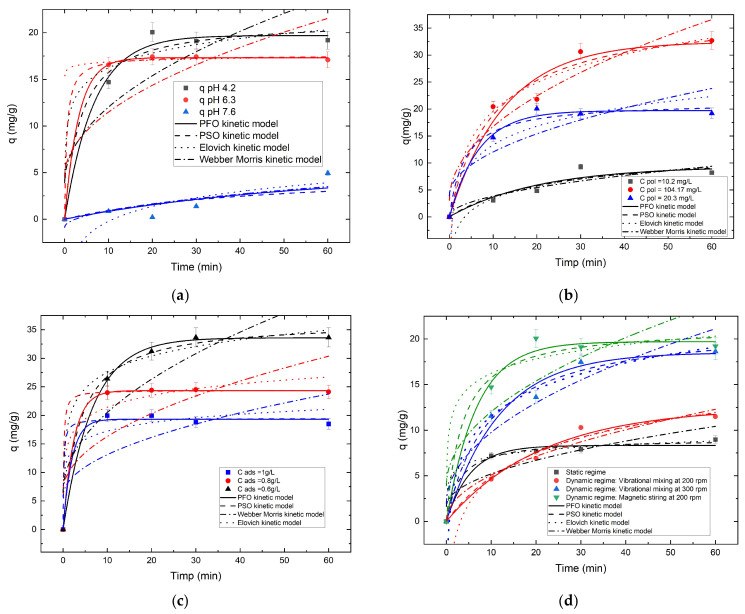
Kinetic modelling of dye uptake capacities profiles obtained at various process paraments investigated: (**a**) pH—at following process conditions: C_dye_ = 20.3 mg/L, C_ads_ = 1.01 g/L, T = 25 °C; Dynamic regime: magnetic stirring at 200 rpm; (**b**) dye concentrations at following process conditions: pH 4.2, T = 25 °C, C_ads_ = 1 g/L, C_dye_ = 10–104 mg/L, mixing regime: dynamic, magnetic stirring at 200 rpm; (**c**) adsorbent concentrations (dosage) at following process conditions: pH 4.2, T = 25 °C, C_dye_ = 20.4 mg/L, C_ads_ = 0.6–1 g/L, mixing regime: dynamic, magnetic stirring at 200 rpm; and (**d**) under static vs. dynamic regimes, using magnetic vs. vibrational mixing at various speed (200 rpm vs. 300 rpm) at following process conditions: pH 4.2, T = 25 °C, C_dye_ = 20.4 mg/L, C_ads_ = 1 g/L, mixing regimes: static vs. dynamic, dynamic: magnetic stirring at 200 rpm vs. vibrational at 200 rpm and vibrational mixing at 200 rpm vs. 300 rpm.

Figure 5 displays the kinetic profiles of the experimental data obtained from varying all the process parameters and expressed as uptake capacities vs. time, modelled with common kinetic models: pseudo-first-order kinetic model, pseudo-second-order kinetic model, Elovich kinetic model, and Weber–Morris kinetic model. The fitted parameters are summarised in Table S1. The best fit of the experimental data to the kinetic models was given by the closest value to unity of the adjusted regression coefficient—adjusted R^2^—as a second statistical parameter used to assess the goodness of fit [31,76].

Effect of pH on Adsorption Capacity. The calculated uptake capacities for reactive yellow adsorption onto magnetite at various pH values align well with the experimental data (Figure 5a). The highest capacity is observed at pH 4.2 (ca. 19 mg/g), followed by pH 6.3 (ca. 17 mg/g), and then pH 7.6 (ca. 5 mg/g). This roughly 75% decrease in capacity within a 3 pH unit range (from 4 to 7) likely stems from changes in both the adsorbent surface charge and the reactive yellow functional group charge in solution. Additionally, adsorption seems faster and reaches equilibrium within 20 min at lower pH compared to neutral pH. Kinetic modelling suggests that the adsorption mechanism is predominantly physical at pH values up to neutral, as evidenced by the best fit to the pseudo-first-order kinetic model given by the closer-to-unity values of the adjusted regression factors: adjusted R^2^ = 0.988, 0.999 and 0.699 for pH 4.2, 6.3 and 7.5 respectively.

Effect of Dye Concentration on Adsorption Kinetics. Figure 5b depicts the influence of initial dye concentration (10–104.7 mg/L) on magnetite’s uptake capacity for reactive yellow. The uptake capacity increases with increasing dye concentration, reaching a maximum of 32.9 mg/L at 104.7 mg/L, indicating a tendency towards saturation. Kinetic data for lower dye concentrations (10 and 20 mg/L) fit well with the pseudo-first-order model (adjusted R^2^ = 0.856 and 0.988, respectively), suggesting a physical adsorption mechanism dominated by weak interactions like Van der Waals or H-bonding. In contrast, higher dye concentrations favour the Elovich kinetic model (adjusted R^2^ = 0.973), implying a shift towards chemisorption. Furthermore, a relative equilibrium is reached later (after 40 min) at high dye concentrations compared to the plateau observed after 25 min with a medium concentration (20 mg/L).

Investigating the impact of adsorbent dosage revealed that magnetite uptake capacities increased from approximately 19 mg/g to 32 mg/g as the particle concentration decreased from 1 g/L to 0.6 g/L (with a fixed dye concentration of 20 mg/L) (Figure 5c). This trend suggests that the limited amount of dye becomes distributed over a larger number of available sites as the adsorbent concentration decreases. Consequently, the system approaches saturation at the lowest particle concentration (0.6 g/L).

Kinetic modelling using the pseudo-first-order model yielded excellent fits to the experimental data (adjusted R^2^ = 0.993–0.999), implying a predominantly physical adsorption mechanism, followed by a very similar fit to the pseudo-second-order kinetic model (adjusted R^2^ = 0.992–0.999), which suggests a coexistence of physical and chemical adsorption mechanisms. Furthermore, the adsorption rate constants derived from the kinetic fits demonstrate a clear dependence on particle concentration. Higher particle concentrations resulted in faster adsorption, reaching equilibrium within the first 10–20 min.

Investigating the impact of the mixing regime and mixing type on reactive dye uptake: static vs. dynamic; dynamic magnetic at 200 rpm vs. dynamic vibrational at 200 rpm and dynamic vibrational at 200 rpm vs. dynamic vibrational at 300 rpm, the results revealed that magnetic stirring at 200 rpm yielded the highest equilibrium uptake capacity (19.70 mg/g) under identical experimental conditions (initial dye concentration, adsorbent dosage, pH, and temperature). Keeping the speed identical, at 200 rpm, but changing the type of mixing: magnetic vs. vibrational, the vibrational mixing significantly decreased equilibrium uptake (12.49 mg/g), indicating that the performance dropped of about one-third compared to the magnetic type of mixing. However, increasing vibrational mixing at 300 rpm achieved a similar uptake capacity (18.51 mg/g) to the magnetic mixing performance, but not identical. Thus, if vibrational mixing is to be available in a reactor/treatment plant, then higher speed might be needed to achieve a similar effect as magnetic stirring. All types of dynamic conditions may be considered and appreciated from a process engineering point of view, when decisions on adsorption efficiency, accounting for material uptake capacity and rheology of the reactor/treatment plant, are to be made at specific sites.

The static regime (no mixing) resulted in the lowest equilibrium uptake capacity, i.e., 8.32 mg/g, almost half that of the best dynamic regime, 19.70 mg/g. This likely stems from two factors: limited particle-liquid interface, reducing the number of exposed surface sites for dye adsorption, and potentially less adsorption occurring between particles themselves. Additionally, the static regime reached equilibrium faster (0.18 min^−1^), possibly due to the rapid saturation of the limited available surface sites.

These findings highlight the significant role of proper mixing in enhancing the adsorbent’s capacity to remove reactive dye from aqueous solutions. For pilot-scale or industrial applications, magnetic stirring emerges as the preferred option due to its effectiveness in maintaining a dynamic regime, maximising adsorbent performance, and facilitating easier recovery of the adsorbent for subsequent cycles.

### 3.5. Adsorption Thermodynamics

#### 3.5.1. Adsorption Isotherms

**Figure 6:**
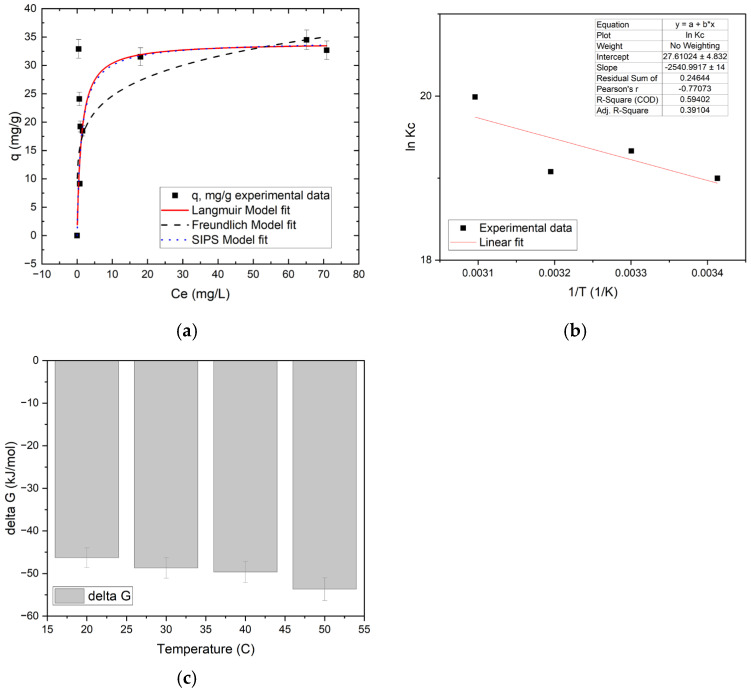
(**a**) Adsorption isotherms of experimental data fitted with Langmuir, Freundlich and Sips models and their appropriate parameters tabulated below. Process conditions: Ci_dye_ = 0–104.17 mg/L, C_ads_ = 1 g/L, dynamic regime, 200 rpm, room temperature, pH 4.2; (**b**) Van `t Hoff plot of experimental data and appropriate linear fit, that allowed thermodynamic parameters ΔHº and ΔSº to be calculated; (**c**) the profile of the change in the Gibss free energy of the reactive yellow adsorption onto magnetite system with temperature.

The experimental data expressed as uptake capacity, q, vs. dye concentration at equilibrium, Ce, were fitted to three isothermal models, in a nonlinear form, to derive empirical information about the adsorption mechanism occurring under a dynamic regime, acidic pH and pollutant concentration ranging up to 104.17 mg/L. The results are displayed in Figure 6, and the summary of fitting parameters is shown in Table 1.

The maximum uptake capacity given by the experimental data is very close to the value calculated by fitting the experimental data to the Langmuir isotherm, approx. 34 mg/g. However, the best fit of the experimental data seems to undertake the SIPS model (adjusted R^2^ = 0.952), suggesting that the adsorption occurs as a combination of mono-layer and multi-layered adsorption at nanoparticle surface sites, under chosen process conditions.

The value of R_L_ is calculated from the Langmuir constant, K_L_, which shows sub unitary values for all dye-working concentrations. This output indicates that the adsorption is favourable.

Langmuir’s weighted value of maximum uptake capacity of magnetite, as an adsorbent, for reactive yellow, as a dye pollutant, derived from isothermal modelling, allows comparison with other nanomaterials tested to be made.

Thus, other iron oxyhydroxides [22], carbonates [21], apatite [77], silicate gels [78] and natural materials [79] such as cotton fibres that were tested for reactive yellow removal, such as magnetite, maghemite or functionalised with ethylene glycol or chitosan or cotton fibres functionalised epichlorohydrin and ammonia water, bare magnetite seem to have good uptake capacity comparable to maghemite doped with ethylene glycol [22] and superior to bare cotton fibres [79].

Among iron oxyhydroxides tested in the literature for the uptake of other types of dyes: such us maghemite tested for Congo Red [80] uptake (q_max_ = 208.33 mg/g) and maghemite functionalised with chitosan tested for Methyl Orange [81] uptake (q_max_ = 29.41 mg/g), at acidic pH values (i.e., pH 2.9 and 5.9, respectively), magnetite uptake for reactive yellow seems superior to maghemite for Methyl Orange and less efficient than maghemite for Congo Red (Table S2).

#### 3.5.2. Changes in the Gibbs Free Energy, Enthalpy and Entropy of the Adsorption

To assess the Gibbs free energy change in the adsorption process or reactive dye onto magnetite, the equilibrium constant was derived from Langmuir fits of experimental data as q_e_ vs. C_e_ (Figure S4) obtained at each temperature, following the Zhou and Zhou (2014) [49] and Tran et al., 2017 [48], as well as Milonjic (2007) [50] recommended approaches.

Table 1 presents the calculated Gibbs free energy change (ΔG°) values for the adsorption process at different temperatures. Negative ΔG° values indicate a spontaneous adsorption process. Interestingly, Figure 4c shows that the magnitude of the negative ΔG° becomes more pronounced with increasing temperature. This suggests that while the adsorption is spontaneous at all the studied temperatures, it becomes increasingly favourable (an increase in system energy) at higher temperatures.

The change in enthalpy (ΔHº) calculated for the reactive dye adsorption onto plain magnetite returned a positive value of 21.12 KJ/mol, indicating that the adsorption process is generally endothermic over the temperature interval studied: it requires energy in the form of heat to occur. However, as temperature increases (over 40), a minor decrease in efficiency (from approx. 100% to approx. 84%, for the lowest-working dye concentration) can be observed, which might suggest a change in system entropy that led to minimal desorption. The change in the entropy (ΔSº) of the current process studied was calculated as 0.22 KJ/(mol × K) after Zhou and Zhou (2014) [49], (or 0.20 KJ/(mol × K) after Milonjic (2007) [50], see Supplementary Information Figure S5). The positive value suggests that the disorder of the system increases with temperature or that the system is becoming more disordered or chaotic. This output may be related to the observation made in the section treating the effect of temperature, stating that an increase in temperature led to an increase in Brownian motion of the system that had a negative effect on adsorbent uptake capacity, potentially explaining light desorption occurring at higher temperatures. This is in accordance with the change in the Gibbs free energy profile, which increased with temperature (see Figure 6c) and indicated an increase in system energy with increasing temperature, as stated above. Also, as |TS°| is greater than |H°| at all the temperatures, it indicates that the adsorption process is dominated by entropic rather than enthalpic changes.

Similar thermodynamic behaviour was found in the literature for phosphate adsorption onto magnetite [67]; for 4-nitrophenole onto magnetic copper ferrite [82], for Reactive Yellow 84 onto silica gel modified with 2,2′-(hexane-1,6-diylbis(oxy)) dibenzaldehyde [78], for the adsorption of lead ions onto *Manihot esculenta* chaff surface showed a decline in adsorption efficiency at 338 K that was attributed to desorption, due to weakening of adsorbent-adsorbate interactions, or structural changes in the raw chaff at elevated temperatures [83] and for the adsorption of reactive brilliant yellow R-4GLN onto carbon-supported metal nickel showed slight desorption at higher temperature due to violent molecular motion, finally suggesting an optimal temperature of 308 K [84].

### 3.6. Mechanistic Information

From the current results on adsorption experimental profiles as a function of pH and knowing that magnetite point of zero surface charge is near 6.5, and also that the large molecule of RY 84 has several functional types of functional groups that are available to bind at nanoparticle surface (by protonation and deprotonation or even dissociation as a function of solution pH) such as: hydroxyl, sulphonyl, chloride and amino, some mechanistic scenarios can be suggested. Under acidic pH the magnetite surface is mainly positively charged (hydroxyl groups are protonated into -OH_2_^+^); thus, negative functional groups of reactive yellow would favour RY binding at the magnetite surface, whereas under neutral to alkaline environments, also above the magnetite PZC value, the nanoparticle surface progressively becomes negatively charged (-O^−^), and so would repeal the large molecule of dye.

**Figure 7:**
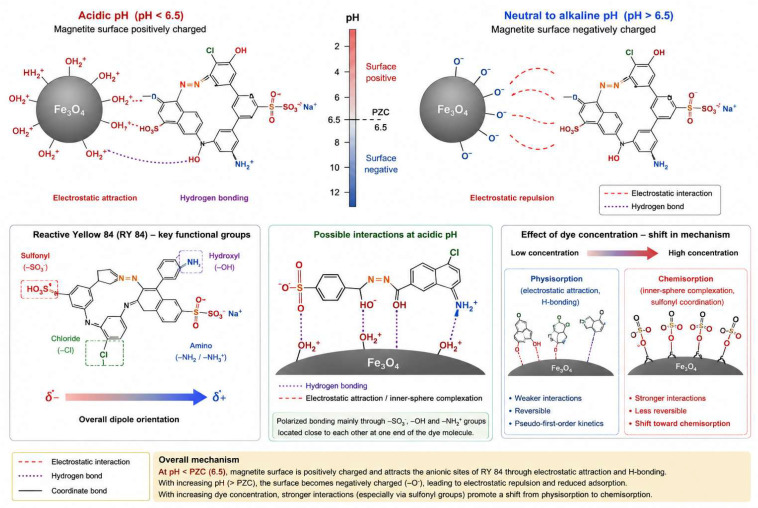
Schematic representation of uptake mechanism showing potential interactions occurring during the RY adsorption process onto magnetite.

A schematic representation of potential interactions occurring during the adsorption process is presented in the Figure 7.

As from overall dipole orientation of the dye molecule (presented in Figure 1d) is likely that polarised bonds at the magnetite surface will occur with hydroxyl, sulphonyl and amino functional groups close to each other at the end of the dye molecular structure. Additionally, at acidic pH, the hydroxyl groups from the dye molecule, as well as protonated sites, -OH_2_^+^ from the magnetite surface, could be involved in forming H-bonding, as the best fit to the PFO kinetic model of the experimental data has indirectly suggested.

As the kinetic and isothermal results empirically indicated, with increasing dye concentration, strong bonding involving sulfonyl groups may occur, justifying a shift in mechanism towards a chemisorption-preponderant one. A combination of physical and chemical adsorption mechanisms coexisting in dye adsorption studies onto various magnetic adsorbents based on zinc and copper ferrite was also found in the literature [45,82].

### 3.7. Magnetite Reuse Tests: Adsorption–Desorption in up to 6 Cycles

**Figure 8:**
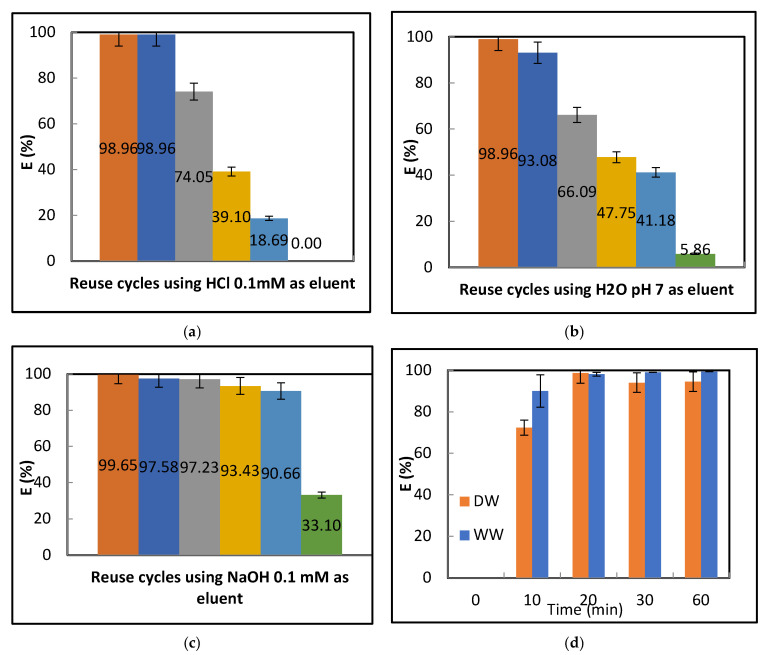
(**a**–**c**): The efficiencies of reactive yellow adsorption onto magnetite reused in six consecutive adsorption–desorption cycles, using as eluent between cycles: (**a**) an acidic solution of HCl 0.1 mM of pH 4, (**b**) a neutral solution of H_2_O pH 7 and (**c**) a basic solution of NaOH 0.1 mM of pH 10. Process conditions: C_dye_ = 20 mg/L, C_ads_ = 1.0 g/L, pH 4.2, T = 20 °C; dynamic regime: magnetic stirring at 200 rpm, contact time 60 min.; (d) Reactive yellow removal efficiency from deionised water *vs.* wastewater, under optimum process conditions: C_dye_ = 20 mg/L, C_ads_ = 1 g/L, pH_dw_ = 4.2, pH_ww_ = 4.1, room temperature, dynamic regime at 200 rpm in simulated batch reactor.

The reusability of magnetite nanoparticles for reactive yellow removal was investigated (Figure 8). Washing the nanoparticles with a mildly acidic solution after each adsorption cycle yielded very good uptake performance (99%) in the second cycle. However, efficiency progressively decreased following each subsequent set of three cycles (dropping to 74.05%, 39.10%, and 18.60%). This trend suggests that while acidic washing offers some regeneration capability, it might not be ideal for long-term reusability beyond three cycles. The desorption profiles (Figure S6b,d,f,h) provide further insights.

These findings highlight the need for further exploration of regeneration techniques that can maintain both the adsorption capacity and the potential degradation ability of magnetite nanoparticles for the efficient removal of reactive yellow over multiple cycles.

Investigating reusability with different eluents revealed contrasting outcomes (Figure 8). Using pure water as an eluent led to a progressive decrease in dye removal efficiency after each cycle, reaching nearly half its initial potential by the fourth cycle (47.75%). This trend aligns with increasing dye desorption observed in Figure S6b,d,f,h, suggesting a mild cleaning effect of water on the magnetite surface, similar to the acidic solution but with less impact. Interestingly, employing a basic solution (pH 10) as the eluent yielded significantly better reusability (Figure 8c). Here, over 90% of the adsorption efficiency was maintained for the first five cycles, with a gradual decrease thereafter. The basic solution also facilitated progressive desorption of the dye, with the desorbed amount increasing from 18.40% in cycle 1 to 85% in cycle 5 (Figure S6b,d,f,h). This behaviour suggests a substantial change in the adsorption–desorption mechanisms compared to systems using acidic or neutral eluents, potentially due to interactions between the basic solution and the magnetite surface.

A potential mechanism for the superior performance of the basic eluent (pH 10) is proposed. The addition of OH^−^ ions might not only regenerate the magnetite surface but also functionalise it. This could facilitate the binding of more reactive yellow functional groups to the hydroxylated surface sites. Additionally, the generation of new hydroxyl radicals (·OH) through a Fenton-like process could be enhanced, promoting further dye degradation. Overall, the reusability tests suggest that the basic solution is the most promising eluent for magnetite regeneration due to its ability to both clean and potentially functionalise the surface. Current research on maghemite nanoparticles for reactive yellow removal has shown promising performance for up to five cycles. Notably, maghemite exhibited a substantially higher reusability of up to eight cycles when using the same adsorbent dosage, half the initial dye concentration (10 mg/L), and a concentrated NaOH solution (1 mM) as the eluent [22].

### 3.8. Adsorption Efficiency in Real Wastewaters

Figure 8d depicts the adsorption profile of reactive yellow onto magnetite in real wastewater. The profile closely resembles that observed in deionised water, suggesting minimal influence of potentially competitive ions from the wastewater matrix on the adsorption process. Interestingly, the initial adsorption appears slightly faster in wastewater compared to deionised water. This might be attributed to the formation of complexes between the dye and certain wastewater components, potentially enhancing the affinity of reactive yellow for the magnetite surface. This finding stands out in contrast to previous studies on reactive yellow removal using calcium carbonate microparticles [21] and maghemite nanoparticles [22], which reported significant efficiency drops (80% to 40%).

### 3.9. Magnetite Nanoparticles’ Uptake Performance for Reactive Yellow Comparison with Other Materials Tested in the Literature

A direct comparison with other literature studies is subjective due to other process conditions and surface properties (such as surface charge properties and pH (that also controls the speciation of dyes in solution, surface area and particle size) that might differ. However, accounting for the above, a trial comparison of the performance of the current 8–25 nm range in size magnetite nanoparticles with other materials tested to remove RY84 from solution is intended. Among few studies that have used various materials to remove this specific dye: Hydroxyapatite: (63 μm, SA = 137 m^2^/g, q = 50.25 mg/g, C_i RY84_ = 10–40 mg/L, pH 5) [77], *vs.* maghemite EG (50 nm, SA = 79.51 m^2^/g, q = 39.42 mg/g, C_i RY84_ = 10–100 mg/L, pH 4) [22], vs. calcium carbonate-EDTA 10% (5 μm, SA = 11.16 m^2^/g, q = 39.35 mg/g, C_i RY84_ = 5–60 mg/L, pH 8) [21] *vs.* Maleate-alumoxane nanoparticle (SA= 99.83 m2/g, q_max_ = 243.9mg/g, Ci _RY84_ = 151.5mg/L, pH 4 [85]) *vs.* current magnetite nanoparticles (8–25 nm range in size with SA = 84.98 m^2^/g, q = 32.399 mg/g, C_i RY84_ = 100–100 mg/L, pH 4.2), it can be noted that magnetite nanoparticles has similar performance to maghemite nanoparticles under acidic pH conditions. Although hydroxyapatite had slightly better uptake capacity (q = 50.25 mg/g) under acidic environments, pH 5, compared to magnetite and maghemite nanoparticles, the latter present the advantage of being easily recoverable and reused in multiple adsorption and desorption cycles. With a similar uptake capacity, 39.35 mg/g, calcium carbonate particles doped with EDTA, despite higher particle size and lower SA, seem to perform comparably to reactive yellow uptake, but under alkaline conditions.

## 4. Conclusions

This study explored the performance of plain and not engineered and easily prepared magnetite nanoparticles for reactive yellow removal, investigating the influence of various process parameters. After a rigorous characterisation of the nanoparticles, the effects of pH, temperature, dye concentration, adsorbent dosage, mixing regime, and speed on both the nanoparticles’ uptake capacity and their ability to remove the dye were evaluated.

### 4.1. Key Findings on the Effect of Process Parameters, Kinetics and Thermodynamic Considerations: Mechanistic Insights

Magnetite performed best under slightly acidic to neutral pH (4–6) conditions. While its uptake capacity decreased with increasing temperature (32.3 mg/g at 20 °C to 17.4 mg/g at 50 °C), it rose with initial dye concentration (from 9.4 mg/g at 10.2 mg/L to 32.5 mg/g at 100 mg/L), reaching saturation near 34 mg/g. The adsorption process demonstrated high efficiency at low dye concentrations (<50 mg/L), achieving over 85% removal within 30 min. At higher concentrations (50–100 mg/L), the removal efficiency was 30–80%. For higher dye concentrations, maintaining feasibility may require increasing the adsorbent dosage (more than 1 g/L). Saturation likely occurs at dye concentrations in aqueous solution between 50 and 100 mg/L under optimal pH (4) and temperature conditions (20 °C).

Kinetic modelling suggested a primarily physical adsorption mechanism (from best fit to pseudo-first-order kinetic model) at low to medium dye concentrations. This transitioned towards chemisorption at higher concentrations (100 mg/L). The choice of mixing regime significantly impacted performance. Dynamic mixing (magnetic stirring) considerably enhanced both uptake capacity and removal efficiency compared to static conditions. This method offers the additional benefit of facilitating nanoparticle recovery for reuse, in up to five adsorption cycles due to their magnetic properties.

Thermodynamic analysis revealed that the reactive dye adsorption onto magnetite is a spontaneous (negative ΔG° values: ΔGº = −46.28, −48.70, −49.65, −53.68 KJ/mol) and endothermic (ΔHº = 21.12 KJ/mol) process. Increasing temperature likely promotes disorder at the adsorbent surface, potentially leading to slight desorption after extended contact times (>60 min), or due to increasing temperature subsequently increasing entropy due to an increase of Brownian motion at the solid interface, which led to slight desorption, or negatively impacting uptake capacity when saturation is reached.

### 4.2. Reuse and Real-World Applicability

Reusability tests demonstrated that magnetite nanoparticles retained high efficiency (>90% removal) for up to 5 cycles when employing a mild alkaline solution (0.1 mM NaOH) as an eluent for dye desorption and surface regeneration. Notably, tests conducted in real wastewater yielded performance comparable to that in simple aqueous solutions, suggesting promise for pilot and industrial-scale applications. 

### 4.3. Future Directions

Future research could explore strategies to optimise the regeneration process for extended reusability. Additionally, investigating potential interactions between magnetite nanoparticles and other wastewater components would provide a more comprehensive understanding of real-world performance.

## 5. Patent

Relevant patent disclosure includes EP 3639020 B1 [86], Separation method using an ion exchanger and a draw solution comprising adsorber particles, inventor: Imad Ahmed, published 14 May 2025. The related patent family includes WO2018229031A1. The patent family relates to separation methods involving ion exchangers, draw solutions, adsorber particles, magnetic particles, adsorption, magnetic separation and elution. The present manuscript reports experimental adsorption, regeneration and wastewater-applicability data for reactive dye removal using magnetite nanoparticles. This disclosure is made for transparency and proper attribution. It does not grant, transfer, waive or limit any intellectual property rights held by Imad Ahmed or Nanolyse Technologies Ltd. Any future technical development, patent filing, licensing or commercialisation activity relating to magnetic nanomaterials, adsorbent systems, regeneration processes or environmental and water-treatment applications will be assessed separately.

## Figures and Tables

**Table 1:** Summary of the adsorption thermodynamic parameters.

Adsorption Isotherm Fitting Parameters to Langmuir, Freundlich and SIPS Isothermal Models.					
	Langmuir Isotherm	Freundlich Isotherm	SIPS Isothermal Model		
Magnetite					
Parameters	q_max_ = 34.02 mg/g K_L_ = 0.829 ± 0.215 R_L_ = 0.056–0.107	K_F_ = 16.25 mg/g 1/*n* = 0.180 ± 0.041	q_max_ = 34.356 ± 1.879 Ks = 0.804 ± 0.203 *n* = 1.077		
Statistics	Adj. R^2^ = 902 R^2^ = 0.920	Adj. R^2^ = 0.819 R^2^ = 0.855	Adj. R^2^ = 0.952 R^2^ = 0.960		
Experimental conditions: C_dye_ = 0 to 104 mg/L, dynamic batch adsorption at pH 4.2, RT, C_ads_ = 1 g/L					
**Thermodynamic parameters for reactive yellow adsorption onto magnetite in aqueous solution**					
**Temp**	**Kc × 10^8^**	**ΔGº, KJ/mol**	**ΔHº, KJ/mol**	**ΔSº, KJ/(mol × K)**	**TS**
20 °C/293 K	1.78	−46.28	21.12	0.229	67.097
30 °C/303 K	2.48	−48.70			69.387
40 °C/313 K	1.93	−49.65			71.677
50 °C/323 K	4.80	−53.68			73.967

With K_L_ to Kc unit conversion after Zhou and Zhou, 2014 [49] and Tran et al., 2017 [48].

## Data Availability

Data supporting the findings of this study are available from the corresponding author upon reasonable request. Full public deposition of the complete dataset and associated technical details is not made at this stage because the authors and relevant organisations may undertake further technical validation, optimisation and intellectual-property assessment relating to magnetic nanomaterial synthesis, regeneration and water-treatment applications.

## Supplementary Materials

The following supporting information can be downloaded at https://www.mdpi.com/article/10.3390/nano16130821/s1. Supplementary information for the Methods Section: Mathematical expressions of kinetic models used to fit the experimental data in this study; Supplementary information for the Results Section: Figure S1. Fourier-Transform Infra-Red (a), Raman (b) and XRD (c) characterisation of magnetic nanoparticles used in the current study; Figure S2. (a) LCF Outputs from absorption interval: −20 eV before the absorption edge and 150 eV post absorption edge: 0.722 weight alike Fe_2_O_3_—representative of Fe(III) and 0.278 weight alike FeO—representative of Fe(II); (b) LCF Outputs from absorption pre edge interval -15 to 0: 0.739 weight alike Fe_2_O_3_—representative of Fe(III) and 0.261 weight alike FeO—representative of Fe(II); (c) LCF Outputs from around absorption edge interval -20—50 eV: 0.714 weight alike Fe_2_O_3_—representative of Fe(III) and 0.286 weight alike FeO—representative of Fe(II), of total weight of 1; Figure S3. Kinetic profiles of the reactive dye removal efficiency (a,c,e) from aqueous solutions by magnetite and magnetite uptake capacities (b,d,f) for reactive yellow at various temperatures and initial dye concentrations of 20 mg/L (a and b), 50 mg/L (c and d) and 100 mg/L (e and f). Process conditions: temperature range: 20–50 °C, C_i dye_ range: 20–100 mg/L. C_ads_ = 1 g/L, pH 4.2, under dynamic regime with magnetic mixing speed of 200 rpm; Figure S4. Adsorption isotherm obtained at different temperatures and used for thermodynamic parameters to be calculated. Process conditions: C_i dye_ 20–100 mg/L. C_ads_ = 1 g/L, pH 4.2, under dynamic regime with magnetic mixing speed of 200 rpm; Figure S5. Van `t Hoff plot of experimental data of reactive yellow adsorption onto plain magnetite and appropriate linear fit, which allowed thermodynamic parameters ΔHº and ΔSº to be calculated; Figure S6. The efficiency of reactive yellow adsorption onto magnetite reused in six consecutive adsorption–desorption cycles, using as eluent between cycles: (a) an acidic solution of HCl 0.1 mM of pH 4, (b) a neutral solution of H_2_O pH 7 and (c) a basic solution of NaOH 0.1 mM of pH 10. Adsorption process conditions: C_dye_ = 20 mg/L, C_ads_ = 1.0 g/L, pH 4.2, T = 20 °C; dynamic regime: magnetic stirring at 200 rpm, contact time 60 min; desorption process conditions: C_eluent_ = 0.1 mM HCl; 0.1 mM NaOH and DI H_2_O brought to pH 7 with NaOH 1 mM, pH 4, 7 and 10, respectively, C_ads_ = 1.0 g/L, T = 20 °C; dynamic regime: magnetic stirring at 200 rpm, contact time 10 min; Table S1. Summary of kinetic fitting parameters of dye adsorption onto magnetite, using pseudo-first-order (PFO), pseudo-second-order (PSO), Elovich and Weber–Morris kinetic models; Table S2. Maximum adsorption capacities of different adsorbents tested for dye removal (after Brinza et al., 2022 [21] and Maftei et al., 2023 [22]); Table S3. Physical and chemical characteristics of wastewater used in this research.

## References

[1] Naim M.M., Al-harby N.F., El Batouti M., Elewa M.M. (2022). Macro-Reticular Ion Exchange Resins for Recovery of Direct Dyes from Spent Dyeing and Soaping Liquors. Molecules.

[2] Singh A.L., Chaudhary S., Kumar S., Kumar A., Singh A., Yadav A. (2022). Biodegradation of Reactive Yellow-145 azo dye using bacterial consortium: A deterministic analysis based on degradable Metabolite, phytotoxicity and genotoxicity study. Chemosphere.

[3] Srinivasan S., Bankole P.O., Sadasivam S.K. (2022). Biodecolorization and degradation of textile azo dyes using *Lysinibacillus sphaericus* MTCC 9523. Front. Environ. Sci..

[4] Topare N.S., Bokil S.A. (2021). Adsorption of textile industry effluent in a fixed bed column using activated carbon prepared from agro-waste materials. Mater. Today Proc..

[5] Yaseen D.A., Scholz M. (2019). Textile dye wastewater characteristics and constituents of synthetic effluents: A critical review. Int. J. Environ. Sci. Technol..

[6] Adegoke K.A., Bello O.S. (2015). Dye sequestration using agricultural wastes as adsorbents. Water Resour. Ind..

[7] Nidheesh P.V., Zhou M., Oturan M.A. (2018). An overview on the removal of synthetic dyes from water by electrochemical advanced oxidation processes. Chemosphere.

[8] McYotto F., Wei Q., Macharia D.K., Huang M., Shen C., Chow C.W.K. (2021). Effect of dye structure on color removal efficiency by coagulation. Chem. Eng. J..

[9] Coromelci C.G., Maftei A.E., Neamtu M., Ababei G., Brinza L. (2024). Amorphous iron oxyhydroxides nano precursors used for Reactive Yellow 84 removal from aqueous solutions. Sep. Purif. Technol..

[10] Coromelci C., Ignat M., Sacarescu L., Neamtu M. (2022). Enhanced visible light activated mesoporous titania by rare earth metal doping. Microporous Mesoporous Mater..

[11] Brinza L. (2022). Surface Coverage Simulation and 3D Plotting of Main Process Parameters for Molybdenum and Vanadium Adsorption onto Ferrihydrite. Nanomaterials.

[12] Zhang W., Ma X., Li R., Yang W., Li Q., Sun X., Li J., Shen J. (2021). Rapid sequestration of chelated Cr(III) by ferrihydrite: Adsorption and overall transformation of Cr(III) complexes. Colloids Surf. A Physicochem. Eng. Asp..

[13] Larsson M.A., Persson I., Sjöstedt C., Gustafsson J.P. (2017). Vanadate complexation to ferrihydrite: X-ray absorption spectroscopy and CD-MUSIC modelling. Environ. Chem..

[14] Lee S., Xu H. (2019). One-Step Route Synthesis of Siliceous Six-Line Ferrihydrite: Implication for the Formation of Natural Ferrihydrite. ACS Earth Space Chem..

[15] Brinza L. (2010). Interactions of Molybdenum and Vanadium with Iron Nanoparticles [Electronic Resource]. http://etheses.whiterose.ac.uk/1082/.

[16] Brinza L., Vu H.P., Neamtu M., Benning L.G. (2019). Experimental and simulation results of the adsorption of Mo and V onto ferrihydrite. Sci. Rep..

[17] Brinza L., Vu H.P., Shaw S., Mosselmans J.F.W., Benning L.G. (2015). Effect of Mo and V on the Hydrothermal Crystallization of Hematite from Ferrihydrite: An in Situ Energy Dispersive X-ray Diffraction and X-ray Absorption Spectroscopy Study. Cryst. Growth Des..

[18] Dzieniszewska A., Kyziol-Komosinska J., Pajak M. (2020). Adsorption and bonding strength of chromium species by ferrihydrite from acidic aqueous solutions. PeerJ.

[19] Toutounchi S., Shariati S., Mahanpoor K. (2019). Synthesis of nano-sized magnetite mesoporous carbon for removal of Reactive Yellow dye from aqueous solutions. Appl. Organomet. Chem..

[20] Nascimento J.R., Bezerra K.C.H., Martins T.D., Carrilho E., Rodrigues C.D., Labuto G. (2021). Textile effluent treatment employing yeast biomass and a new nanomagnetic biocomposite. Environ. Sci. Pollut. Res..

[21] Brinza L., Maftei A.E., Tascu S., Brinza F., Neamtu M. (2022). Advanced removal of Reactive Yellow 84 azo dye using functionalised amorphous calcium carbonates as adsorbent. Sci. Rep..

[22] Maftei A.E., Ahmed I., Neamtu M., Coromelci C.G., Ignat M., Brinza L. (2023). Nanocrystalline structured ethylene glycol doped maghemite for persistent pollutants removal. Environ. Sci. Water Res. Technol..

[23] Momma K., Izumi F. (2011). VESTA 3 for three-dimensional visualization of crystal, volumetric and morphology data. Appl. Crystallogr..

[24] Bergwerf Labs (2024). MolView.

[25] Vaitkus A., Merkys A., Sander T., Quirós M., Thiessen P.A., Bolton E.E., Gražulis S. (2023). A workflow for deriving chemical entities from crystallographic data and its application to the Crystallography Open Database. J. Cheminform..

[26] Kim S., Chen J., Cheng T., Gindulyte A., He J., He S., Li Q., Shoemaker B.A., Thiessen P.A., Yu B. (2023). PubChem 2023 update. Nucleic Acids Res..

[27] Ravel B., Newville M.A. (2005). ATHENA, ARTEMIS, HEPHAESTUS: Data analysis for X-ray absorption spectroscopy using IFEFFIT. J. Synchrotron Radiat..

[28] (2022). Determination of the Specific Surface Area of Solids by Gas Adsorption—BET Method.

[29] Martínez L.J., Muñoz-Bonilla A., Mazario E., Recio F.J., Palomares F.J., Herrasti P. (2015). Adsorption of chromium(VI) onto electrochemically obtained magnetite nanoparticles. Int. J. Environ. Sci. Technol..

[30] Maftei A.E., Lupu A., Rodriguez-Blanco J.D., Rateau R., Brinza L. (2025). Chromium removal via coprecipitation with carbonates and iron oxyhydroxides minerals: The effect of organic complexing agents. Sci. Total Environ..

[31] Wang J., Guo X. (2020). Adsorption kinetic models: Physical meanings, applications, and solving methods. J. Hazard. Mater..

[32] Ho Y.-S. (2006). Review of second-order models for adsorption systems. J. Hazard. Mater..

[33] Brandani S. (2021). Kinetics of liquid phase batch adsorption experiments. Adsorption.

[34] Qiu H., Lv L., Pan B.-c., Zhang Q.-j., Zhang W.-m., Zhang Q.-x. (2009). Critical review in adsorption kinetic models. J. Zhejiang Univ. -Sci. A.

[35] Mahapatra U., Manna A.K., Chatterjee A. (2022). A critical evaluation of conventional kinetic and isotherm modeling for adsorptive removal of hexavalent chromium and methylene blue by natural rubber sludge-derived activated carbon and commercial activated carbon. Bioresour. Technol..

[36] Ho Y.S., McKay G. (1998). A Comparison of Chemisorption Kinetic Models Applied to Pollutant Removal on Various Sorbents. Process Saf. Environ. Prot..

[37] Elovich S.Y., Larinov O.G. (1962). Theory of adsorption from solutions of non electrolytes on solid (I) equation adsorption from solutions and the analysis of its simplest form, (II) verification of the equation of adsorption isotherm from solutions. Izv. Akad. Nauk. SSSR Otd. Khim. Nauk.

[38] Fila D., Hubicki Z., Kołodyńska D. (2022). Applicability of new sustainable and efficient algi-nate-based composites for critical raw materials recovery: General composites fabrication op-timization and adsorption performance evaluation. Chem. Eng. J..

[39] Lagergren S. (1886). Zur theorie der sogenannten adsorption gelöster stoffe, Kungliga Svenska Vetenskapsakademiens. Handlingar.

[40] Mclintock I.S. (1967). The Elovich Equation in Chemisorption Kinetics. Nature.

[41] Weber W.J., Morris J.C. (1963). Kinetics of adsorption on carbon from solution. ASCE Sanit. Eng. Div. J..

[42] Langmuir I. (1916). The constitution and fundamental properties of solids and liquids. J. Am. Chem. Soc..

[43] Langmuir D. (1997). Aqueous Environmental Geochemistry.

[44] Fang D., Zhuang X., Huang L., Zhang Q., Shen Q., Jiang L., Xu X., Ji F. (2020). Developing the new kinetics model based on the adsorption process: From fitting to comparison and prediction. Sci. Total Environ..

[45] Ahmed M.A., Ahmed M.A., Mohamed A.A. (2022). Facile adsorptive removal of dyes and heavy metals from wastewaters using magnetic nanocomposite of zinc ferrite@reduced graphene oxide. Inorg. Chem. Commun..

[46] Al-Ghouti M.A., Da’ANa D.A. (2020). Guidelines for the use and interpretation of adsorption iso-therm models: A review. J. Hazard. Mater..

[47] (2007). Origin, Version 2023.

[48] Tran H.N., You S.-J., Hosseini-Bandegharaei A., Chao H.-P. (2017). Mistakes and inconsistencies regarding adsorption of contaminants from aqueous solutions: A critical review. Water Res..

[49] Zhou X., Zhou X. (2014). The unit problem in the thermodynamic calculation of adsorption using the langmuir equation. Chem. Eng. Commun..

[50] Milonjić S.K. (2007). A consideration of the correct calculation of thermodynamic parameters of adsorption. J. Serbian Chem. Soc..

[51] Bragg W.H. (1915). The Structure of Magnetite and the Spinels. Nature.

[52] Fjellvåg H., Grønvold F., Stølen S., Hauback B. (1996). On the Crystallographic and Magnetic Structures of Nearly Stoichiometric Iron Monoxide. J. Solid State Chem..

[53] Thommes M., Kaneko K., Neimark V.A., Olivier P.J., Rodriguez-Reinoso F., Rouquerol J., Sing S.W.K. (2015). Physisorption of gases, with special reference to the evaluation of surface area and pore size distribution (IUPAC Technical Report). Pure Appl. Chem..

[54] Thommes M. (2010). Physical Adsorption Characterization of Nanoporous Materials. Chem. Ing. Tech..

[55] Shahid M.K., Choi Y. (2020). Characterization and application of magnetite Particles, synthesized by reverse coprecipitation method in open air from mill scale. J. Magn. Magn. Mater..

[56] Bhole R., Gonsalves D., Murugesan G., Narasimhan M.K., Srinivasan N.R., Dave N., Varadavenkatesan T., Vinayagam R., Govarthanan M., Selvaraj R. (2023). Superparamagnetic spherical magnetite nanoparticles: Synthesis, characterization and catalytic potential. Appl. Nanosci..

[57] Eder S.H., Gigler A.M., Hanzlik M., Winklhofer M. (2014). Sub-micrometer-scale mapping of magnetite crystals and sulfur globules in magnetotactic bacteria using confocal Raman micro-spectrometry. PLoS ONE.

[58] Shebanova O., Lazor P. (2003). Raman study of magnetite (Fe_3_O_4_): Laser-induced thermal effects and oxidation. J. Raman Spectrosc..

[59] Hanesch M. (2009). Raman spectroscopy of iron oxides and (oxy)hydroxides at low laser power and possible applications in environmental magnetic studies. Geophys. J. Int..

[60] Shebanova O., Lazor P. (2003). Raman spectroscopic study of magnetite (FeFe_2_O_4_): A new assignment for the vibrational spectrum. J. Solid State Chem..

[61] Martínez-Matamoros D., Castro-García S., Balado M., Matamoros-Veloza A., Camargo-Valero M.A., Cespedes O., Rodríguez J., Lemos M.L., Jiménez C. (2019). Preparation of functionalized magnetic nanoparticles conjugated with feroxamine and their evaluation for pathogen detection. RSC Adv..

[62] Li Y.-S., Church J.S., Woodhead A.L. (2012). Infrared and Raman spectroscopic studies on iron oxide magnetic nano-particles and their surface modifications. J. Magn. Magn. Mater..

[63] Cornell R.M., Schwertmann U. (2003). The Iron Oxides: Structure, Proprieties, Reactions, Occurances and Uses.

[64] Shebanova O., Lazor P. (2003). Vibrational modeling of the thermodynamic properties of magnetite (Fe_3_O_4_) at high pressure from Raman spectroscopic study. J. Chem. Phys..

[65] Schwaminger S.P., Bauer D., Fraga-García P., Wagner F.E., Berensmeier S. (2017). Oxidation of magnetite nanoparticles: Impact on surface and crystal properties. CrystEngComm.

[66] Pajak L., Bierska-Piech B., Mrowiec-Bialon J., Jarzębski A., Diduszko R. (2005). SAXS from particle and disordered systems. Fibres Text. East. Eur..

[67] Ajmal Z., Muhmood A., Usman M., Kizito S., Lu J., Dong R., Wu S. (2018). Phosphate removal from aqueous solution using iron oxides: Adsorption, desorption and regeneration characteristics. J. Colloid Interface Sci..

[68] Milonjić S.K., Kopečni M.M., Ilić Z.E. (1983). The point of zero charge and adsorption properties of natural magnetite. J. Radioanal. Chem..

[69] Teshager F.M., Habtu N.G., Mequanint K. (2022). A systematic study of cellulose-reactive anionic dye removal using a sustainable bioadsorbent. Chemosphere.

[70] Weng C.H., Lin Y.T., Tzeng T.W. (2009). Removal of methylene blue from aqueous solution by adsorption onto pineapple leaf powder. J. Hazard. Mater..

[71] Zhang C.C., Ma X.Q., Pan T., Zhang Y., Jiang H.Y., Yao J.B. (2023). Nature dye/nanosphere dispersion with high light stability and colorability for cellulose fabric. Text. Res. J..

[72] Hussein T.K. (2023). Removal of Vat Green 3 Dye from Aqua Solution using Chemical Coagulants and Okra Pods as Natural Coagulant by Coagulation- Flocculation Process. Pollution.

[73] Cigeroglu Z., Hasimoglu A., Özdemir O.K. (2021). Synthesis, characterization and an application of graphene oxide nanopowder: Methylene blue adsorption and comparison between experimental data and literature data. J. Dispers. Sci. Technol..

[74] Akar S.T., Koc E., Sayin F., Kara I., Akar T. (2021). Design and modeling of the decolorization characteristics of a regenerable and eco-friendly geopolymer: Batch and dynamic flow mode treatment aspects. J. Environ. Manag..

[75] Hassani A., Soltani R.D.C., Karaca S., Khataee A. (2015). Preparation of montmorillonite-alginate nanobiocomposite for adsorption of a textile dye in aqueous phase: Isotherm, kinetic and experimental design approaches. J. Ind. Eng. Chem..

[76] Simonin J.-P. (2016). On the comparison of pseudo-first order and pseudo-second order rate laws in the modeling of adsorption kinetics. Chem. Eng. J..

[77] Barka N., Qourzal S., Assabbane A., Nounah A., Ait-Ichou Y. (2011). Removal of Reactive Yellow 84 from aqueous solutions by adsorption onto hydroxyapatite. J. Saudi Chem. Soc..

[78] Banaei A., Samadi S., Karimi S., Vojoudi H., Pourbasheer E., Badiei A. (2017). Synthesis of silica gel modified with 2,2′-(hexane-1,6-diylbis(oxy)) dibenzaldehyde as a new adsorbent for the removal of Reactive Yellow 84 and Reactive Blue 19 dyes from aqueous solutions: Equilibrium and thermodynamic studies. Powder Technol..

[79] Jóźwiak T., Filipkowska U., Brym S., Zyśk M. (2020). The use of aminated cotton fibers as an unconventional sorbent to remove anionic dyes from aqueous solutions. Cellulose.

[80] Afkhami A., Moosavi R. (2010). Adsorptive removal of Congo red, a carcinogenic textile dye, from aqueous solutions by maghemite nanoparticles. J. Hazard. Mater..

[81] Jiang R., Fu Y.-Q., Zhu H.-Y., Yao J., Xiao L. (2012). Removal of methyl orange from aqueous solutions by magnetic maghemite/chitosan nanocomposite films: Adsorption kinetics and equilibrium. J. Appl. Polym. Sci..

[82] Ahmed M.A., Ahmed M.A., Mohamed A.A. (2023). Removal of 4-nitrophenol and indigo carmine dye from wastewaters by magnetic copper ferrite nanoparticles: Kinetic, thermodynamic and mechanistic insights. J. Saudi Chem. Soc..

[83] El-Rayyes A., Arogundade I., Ofudje E.A., Refat M.S., Alsuhaibani A.M., Akande J.A., Sodiya E.F. (2025). Thermodynamic, isotherm and kinetic studies lead ions adsorption onto Manihot esculenta chaff surface. Sci. Rep..

[84] Lv B., Xu J., Kang H., Liang P., Wang W., Tao F. (2022). Adsorption Behavior of Magnetic Carbon-Supported Metal Nickel for the Efficient Dye Removal from Water. Int. J. Env. Res. Public Health.

[85] Ghabaee S., Behin J., Ansari M., Rajabi L. (2020). Synthesis and characterization male-ate-alumoxane nanoparticles for removal of reactive yellow 84 dye from aqueous solution. Adv. Powder Technol..

[86] Ahmed I. (2025). Separation Method Using an Ion Exchanger and a Draw Solution Comprising Adsorber Particles. JUSTIA Patent.

