# Supplementary material for: Application of Magnetic Nanoparticles for Reactive Dye Removal from Aqueous Solutions: Practical and Theoretical Approaches
Source: Nanomaterials (Basel). 2026 Jul 2;16(13):821. doi: 10.3390/nano16130821 (PMC13362606; doi:10.3390/nano16130821)
Supplement: Supplementary file 1 [file nanomaterials-16-00821-s001.zip › nanomaterials-4345634-supplementary.pdf]

Application of Magnetic Nanoparticles for Reactive Dye Removal from Aqueous Solutions: Practical and Theoretical Approaches

Iuliana Gabriela Breaban [1], Imad A. M. Ahmed [2,3], Maria Ignat [4] and Loredana Brinza [1,*]

[1] Integrated Center for Environmental Science Studies for the North-East Development Region (CERNESIM), Institute of Interdisciplinary Research / Department of Geography, Faculty of Geography and Geology, Alexandru Ioan Cuza University of Iasi, 11, Carol I Bvd,
700506, Iași, Romania; iulianab@uaic.ro

[2] Department of Earth Sciences, University of Oxford, South Parks Road, Oxford OX1 3AN, UK;

[3] Nanolyse Technologies Ltd , Rutherford Appleton Laboratory, R27 Atlas Centre, Harwell Campus, Didcot OX11 0QX, UK, imad.ahmed@nanolyse.com

[4] Faculty of Chemistry, Alexandru Ioan Cuza University of Iași, 11, Carol I Bvd, 700506, Iași, Romania;
maria.ignat@uaic.ro

* Correspondence: loredana.brinza@uaic.ro; Tel.: +40-0232-202395

Supplementary information

1. Materials and methods

1.1. Adsorption kinetics modelling

The literature contains various kinetic models that are used to interpret absorption mechanisms [1, 2], of which here, four as the most common, were selected to fit the experimental data of this study. For the adsorption kinetic modeling studies, the Pseudo First Order (PFO), the Pseudo Second Order (PSO), and the Elovich and the Weber Morris kinetic models [1-4] were used. The best fit to one of the kinetic models enabled adsorption rates and weighted maximum adsorption uptake capacities to be compared under various conditions and also offer empirical mechanistic information about adsorption.

Adsorption is a complex process that takes place in a system at solid–liquid interface. At the microscale to nanoscale view, it is considered to occur as: (i) the adsorbate transfer from solution towards the adsorbent surface sites and (ii) the main binding process of the adsorbate ions/molecules at the adsorbent surface sites given by various functional groups. The adsorbate transfer from solution to an adsorbent surface may be influenced by the resistance of a few boundaries such as the diffuse film of liquid that surrounds the particles also so-called film (external) diffusion; followed by surface diffusion which is the dense liquid film closed to sorbent surface and finally pore diffusion if the adsorbent surface is porous. Any of the above steps might, preponderantly, influence the sorption process, determining the adsorption speed. Understanding which of these steps mostly affects the sorption process has a significant role in the adsorption design as well as its optimization from the process engineering point of view.

Among the most used models that are used to identify and differentiate the nature of the process of adsorbate binding at the adsorbent surface sites the PFO and PSO are mainly used[2]. Briefly, here, a best fit to the PFO kinetic model implies a mainly physical adsorption mechanism (based on weak interactions such as hydrogen or Van der Walls bonds) whereas a best fit to the PSO kinetic model would indicate a chemical sorption mechanism (based on strong covalent bonds). It is also accepted that the PFO kinetic model assumes that the rate of pollutant sorption is proportional to the number of vacant sites on the sorbent, whereas the PSO kinetic model assumes that the rate of pollutant sorption is proportional to the square of the number of vacant sites on the sorbent [4, 5].

Models such as Weber Morris are used to identify and differentiate among the limiting step that mainly drives the adsorption process. Moreover, if the experimental data is the best fit to the Weber-Morris model and the fitting passes through the origin of the coordinate system, the adsorption process is controlled by intraparticle diffusion. On the other hand, if the fit is composed of multiple lines the adsorption process is affected by multiple mechanisms, such as film diffusion or surface adsorption [1, 6].

The Elovich equation has been extensively applied to chemisorption data for various pollutants uptake from gases and wastewater. It was first proposed by Roginsky and Zeldovich in 1934 [7, 8] and

the equation is presented below. The Elovich Adsorption Kinetic Model is an empirical rate equation that states that the adsorption energy rises in a linear relationship with surface coverage. The model assumes that adsorption occurs at localized sites, and the interaction between adsorbed ions is present, for systems where the concentration of adsorbate is considered to be constant.

Mathematical expressions of kinetic models used to fit the experimental data in this study		
Kinetic model	Equation	References
Pseudo first-order model	Nonlinear form: $\frac{dq_t}{dt} = k_1 \times (q_e - q_t)$<br>Linear form: $\ln(q_e - q_t) = \ln q_e - k_1 \times t$<br>$k_1 = pseudo\ first\ order\ kinetic\ rate\ cofficient, min^{-1}$	[9]
Pseudo second order model	Nonlinear form: $\frac{dq_t}{dt} = k_2 \times (q_e - q_t)^2$<br>Linear form: $\frac{1}{q_t} = \frac{1}{k_2 \times q_e^2} + \frac{1}{q_e} \times \mathrm{t}$<br>$k_2 = pseudo\ second\ order\ kinetic\ rate\ cofficient, g\ mg^{-1}min^{-1}$	[3, 4, 10]
Elovich model	$\frac{dq_t}{dt} = \beta e^{-\alpha q}$<br>*q = amount of element chemisorbed at time t, α is adsorption rate and β is a constant.*	[7]
Weber-Morris interparticle diffusion model	$q_t t = K_{id}\ t^{0.5} + C$<br>$K_{id}$ is the intra-particle diffusion rate constant (mg/g $min^{1/2}$); C is the initial adsorption (mg/g);	[11]

1.2. Adsorption isotherms

Langmuir model is a theoretical model, which assumes that adsorption sites are homogenous, distributed on the adsorbent surface, energetically similar, and that the adsorbate adsorption takes place as monolayers. It accounts for surface coverage by balancing the relative adsorption and desorption rates (dynamic equilibrium). Adsorption is proportional to the fraction of the surface of the open adsorbent surface, while desorption is proportional to the fraction of the covered adsorbent surface [12].

$$q_e = \frac{q_{max} K_L C_e}{1 + K_L C_e}$$

Where q_{max} is the maximum adsorption capacity (mg/g), q_e is the adsorption capacity at equilibrium (mg/g), C_e is adsorbate concentration in solution at equilibrium (mg/L) and K_L is dimensionless Langmuir constant related to the feasibility of adsorption.

Derived from Langmuir isotherms is a so-called separation factor $R_{L,}$ which can be calculated by the following equation.

$$R_L = 1/(1 + K_L\ C_o)$$

Where K_L is Langmuir constant and *Co* is adsorbate initial concentration (mg/L).

The value of R_L assumes the nature and the feasibility of the adsorption process as presented below:

R_L value	Adsorption process
$R_L > 1$	Unfavorable
$R_L = 1$	Linear
$0<R_L < 1$	Favorable
$R_L = 0$	Irreversible

Freundlich isotherm is an empiric model that hypothetically assumes that the adsorption sites are heterogeneously distributed and that the adsorption takes place as a multilayer. It is an expression of surface heterogeneity and the exponential distribution of active sites and their energies [17].

$$q_e = K_f C_e^{1/n}$$

Where q_e is the adsorption capacity at equilibrium (mg/g), C_e is adsorbate concentration in solution at equilibrium (mg/L), K_f is Freundlich constant (mg/g), $1/n$ is Freundlich exponent related to the adsorption intensity and it also indicates the relative distribution of the energy and the heterogeneity of the adsorbent surface sites.

The SIPS isothermal model serves as a hybrid, merging elements from both the Langmuir and Freundlich models, offering a more comprehensive perspective of the adsorption process [13]. The SIPS model is more complex and versatile, as it considers multiple steps and interactions involved in the adsorption process. It is often used when the adsorption behaviour doesn't strictly adhere to a single-layer Langmuir model due to the presence of multilayer adsorption, heterogeneity of the surface, or interactions between adsorbed molecules.

$$q_e = \frac{q_{max}(K_s * C_e)^{\frac{1}{n}}}{1 + (K_s * C_e)^{\frac{1}{n}}}$$

Where,

q e is the equilibrium adsorption capacity (mg/g), q max is the maximum adsorption capacity (mg/g), C_e is the concentration of pollutant at equilibrium (mg/L), K_S is the empirical constant of the Langmuir equation related to energy of adsorption (L/mg), n is the adsorption intensity.

The SIPS isothermal model allows for a more flexible description of adsorption behaviour, accommodating cases where multiple layers of adsorbate are formed on the adsorbent surface or where interactions between adsorbed molecules affect the overall adsorption process. It is particularly useful for systems where the adsorption process is not well-represented by simpler models like the Langmuir or Freundlich isotherms.

2. Results

2.1. Magnetite characterization: spectroscopic and diffraction results

FTIR, Raman spectroscopic and XRD results

(a)

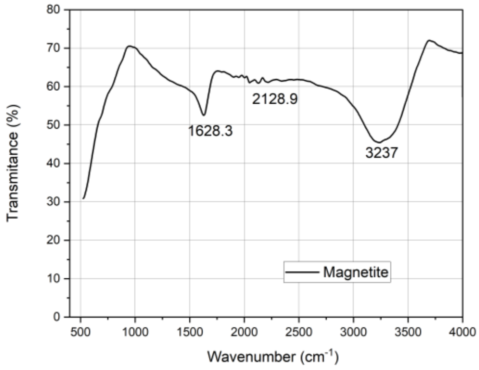

(b)

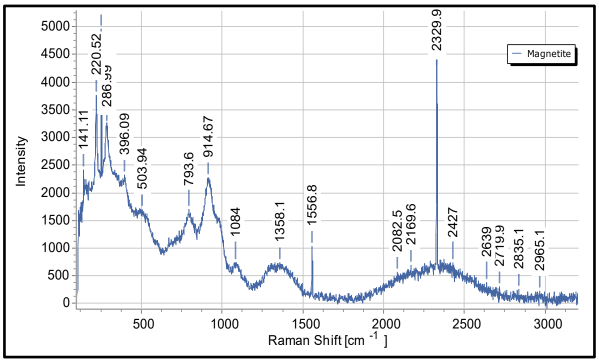

(c)

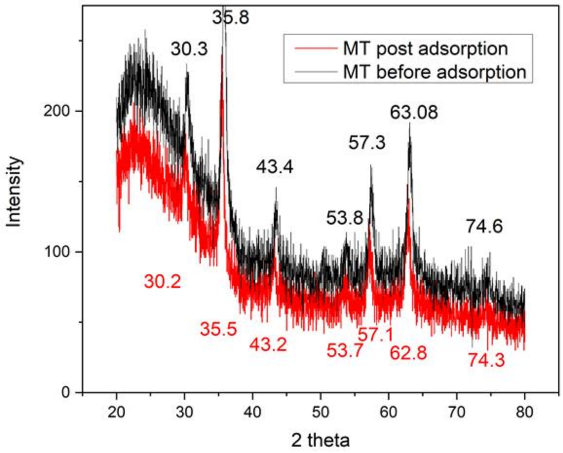

Figure S1. Fourier Transformed Infra-Red (a) Raman (b) and XRD before and post adsorbtion (c) characterization of magnetic nanoparticles used in the current study.

XAS results

Linear combination fitting of Fe-Kα XANES spectra over the fallowing intervals: −20 eV before the absorption edge and 150 eV post absorption edge interval; -15-0 eV pre-edge interval and -20 to 50 eV over the edge interval

(a)

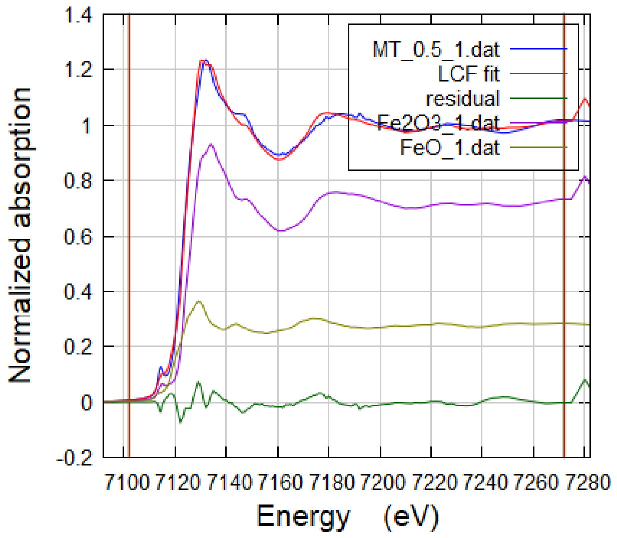

(b)

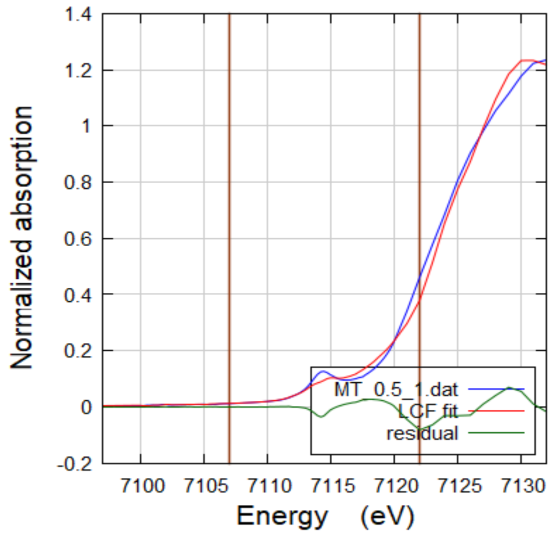

(c)

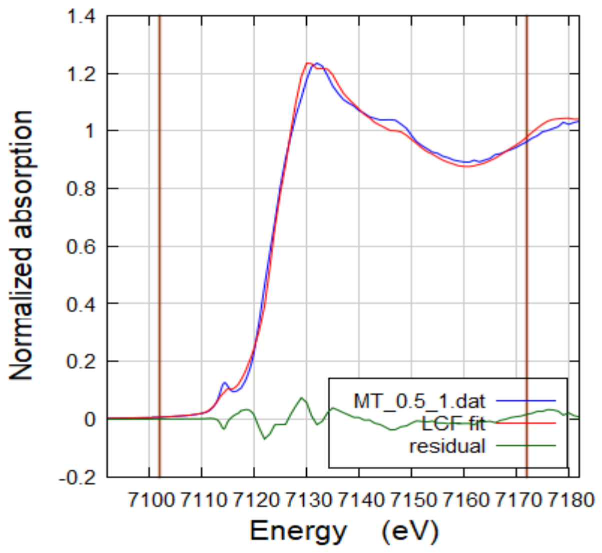

Figure S2. (a) LCF Outputs from absorption interval : −20 eV before the absorption edge and 150 eV post absorption edge: 0.722 weight alike Fe_2O_3 – representative of Fe(III) and 0.278 weight alike FeO – representative of Fe(II); (b) LCF Outputs from absorption pre edge interval -15 to 0: 0.739 weight alike Fe_2O_3 – representative of Fe(III) and 0.261 weight alike FeO – representative of Fe(II); (c) LCF Outputs from around absorption edge interval -20 – 50 eV: 0.714 weight alike Fe_2O_3 – representative of Fe(III) and 0.286 weight alike FeO – representative of Fe(II), of total weight of 1.

2.2. Effect of temperature and initial dye concentration onto adsorption uptake capacity

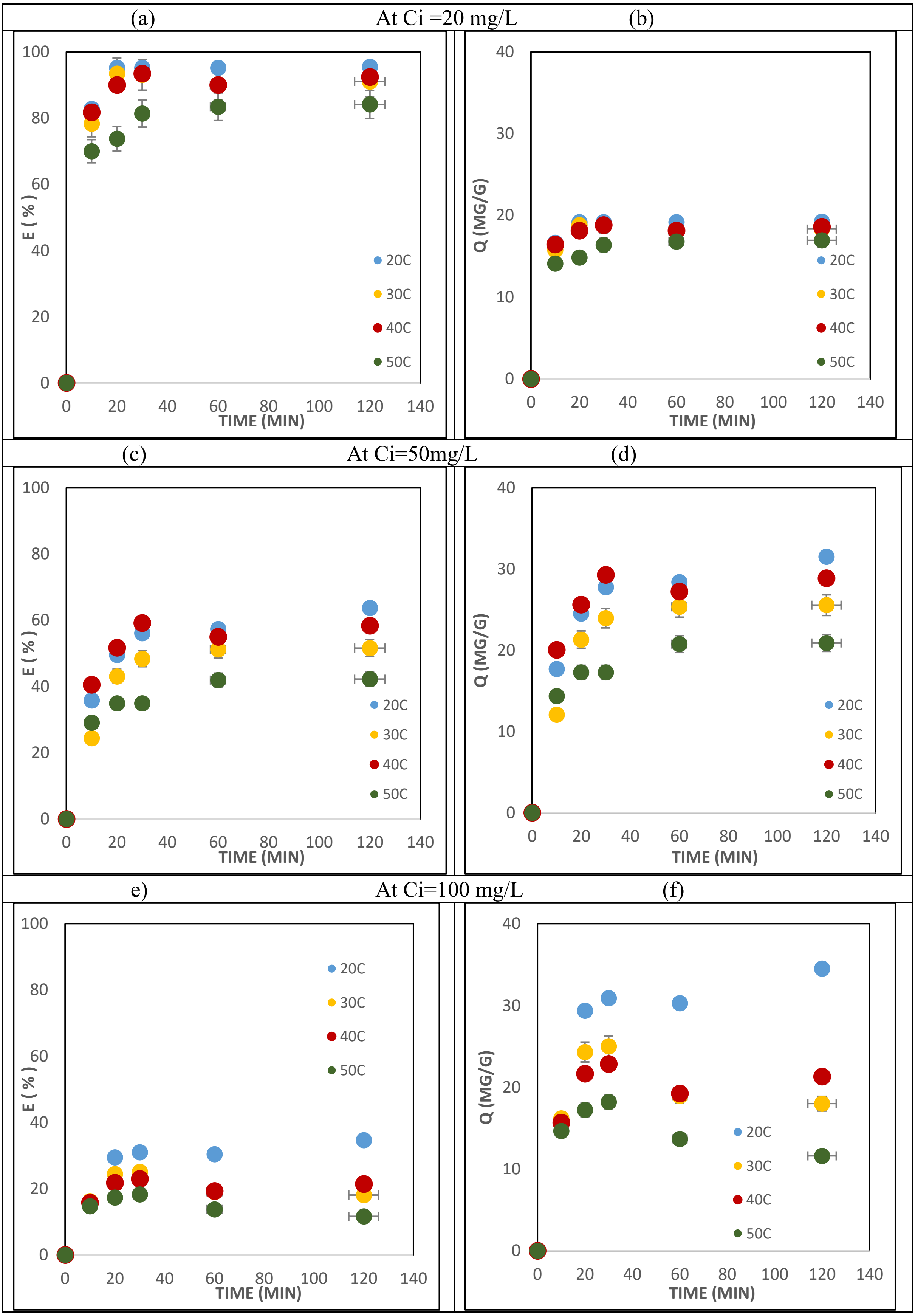

Figure S3. Kinetic profiles of the reactive dye removal efficiency (a,c,e) from aqueous solutions by magnetite and magnetite uptake capacities (b,d,f) for reactive yellow at various temperatures and initial dye concentrations of 20 mg/L (a and b), 50 mg/L (c and d) and 100mg/L (e and f). Process conditions: temperature range: 20-50ºC, Ci dye range: 20-100 mg/L. C ads=1g/L, pH 4.2, under dynamic regime with magnetic mixing speed of 200 rpm.

2.3. Kinetic modeling results

Table S1. Summary of kinetic fitting parameters of dye adsorption onto magnetite, using pseudo-first-order (PFO), pseudo-second-order (PSO), Elovich and Weber Morris kinetic models.

**Value of process parameter tested**	**PFO model**			**PSO model**			**Elovich**			**Weber Morris**		
	**q (mg/g)**	**k_1 (min^{-1})**	**Aj.R^2**	**q (mg/g)**	**k_2 (g $mg^{-1}min^{-1}$)**	**Aj.R^2**	**alpha**	**beta**	**Aj.R^2**	**k**	**C**	**Aj.R^2**
**pH**												
**4.2**	19.700 ±0.650	0.148 ±0.024	**0.988**	21.356 ±1.668	0.013 ±0.007	0.973	286.080 ±0*	0.439 ±0.020	0.969	2.569 ±0.372	3.895 ±0*	0.765
**6.3**	17.321 ±0.100	0.312 ±0.026	**0.999**	17.544 ±0.283	0.114 ±0.068	0.998	4.319 ± 0*	3.4373 ±60.03	0.998	2.193 ±0.398	4.547 ±0*	0.675
**7.5**	5.000 ±0*	0.018 ±0.007	**0.699**	5.285 ±0*	0.004 ±0.002	0.614	0.209 ±0.057	0.432 ±0.175	0.635	0.564 ±0.237	-0.885 ±1.165	0.536
**C adsorbate, (mg/L)**												
**104.17 mg/L**	32.468 ±2.837	0.078 ±0.021	0.950	38.325 ±4.453	0.002 ±0.001	0.961	10.08 ± 0*	0.1321 ±0.007	**0.973**	4.326 ±0.333	3.056 ±0*	0.920
**20.3 mg/L**	19.70 ±0.65	0.149 ±0.025	**0.988**	21.356 ±1.668	0.013 ±0.007	0.973	7.117± 0.388	0.199 ±0.017	0.921	2.57 ±0.372	3.89 ±0*	0.765
**10.2 mg/L**	9.368 ±0.062	0.049 ±0.025	**0.856**	12.637 ± 4.716	0.003 ±0.004	0.834	0.926± 0.388	0.305 ±0.112	0.831	1.205 ±0.140	0.038 ±0*	0.835
**C adsorbent, (mg/L)**												
**0.6 g/L**	33.584 ±0.355	0.151 ±0.008	**0.998**	36.574 ±0.989	0.007 ±0.001	0.996	326.144 ±0*	0.242 ±0.005	0.992	4.453 ±0.579	6.411 ±0*	0.802
**0.8 g/L**	24.327 ±0.098	0.421 ±0.055	**0.999**	24.404 ± 0.204	0.295 ±0.311	0.999	421.18 ±0*	0.339 ±0.01	0.969	3.069 ±0.576	6.582 ±0*	0.661
**1 g/L**	19.329 ±0.348	0.504 ±0*	**0.993**	19.435 ±0.367	0.363 ± 0*	0.992	698.57 ±0*	0.469 ±0.027	0.946	2.318 ±0.504	5.766 ±0*	0.591
**Mixing regime and type**												
**Static regime**	8.322 ±0.329	0.187 ±0.047	0.980	9.049 ±0.369	0.038 ±0.013	0.992	150.004 ±0*	1.037 ±0.011	**0.998**	1.135 ±0.142	1.624 ±0*	0.813
**Vibrational 200 rpm**	12.490 ±1.001	0.046 ±0.008	**0.979**	16.717 ±2.388	0.002 ±0.001	0.973	1.294 ±0.243	0.247 ±0.037	0.970	1.573 ±0.083	0.101 ±0*	0.960
**Vibrational 300 rpm**	18.514 ±1.002	0.084 ±0.014	0.979	21.756 ±1.499	0.004 ±0.001	0.985	6.496 ±0*	0.236 ±0.008	**0.988**	2.48 ±0.19	1.907 ±0*	0.921
**Magnetic 200 rpm**	19.700 ±0.650	0.1489 ±0.024	**0.988**	21.355 ±1.668	0.013 ±0.007	0.973	286.08 ±0*	0.439 ±0.020	0.969	2.569 ±0.372	3.894 ±0*	0.765
*fixed parameters												

2.4. Adsorption thermodynamics

Determination of K_L for experimental set designed for investigation the adsorption thermodynamics and the effect of temperature on adsorption process.

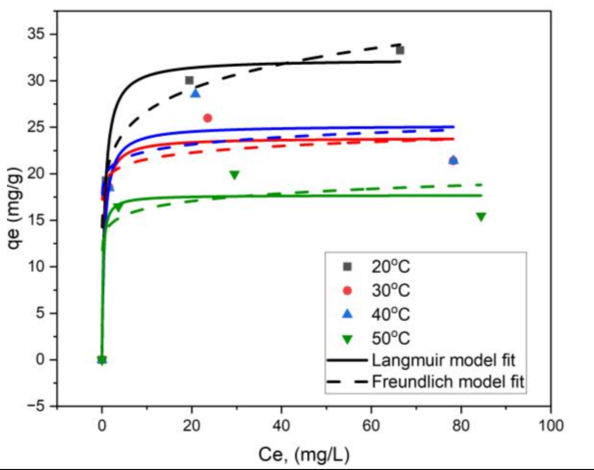

Langmuir adsorption isotherm fitting parameters used to calculate equilibrium constant for thermodynamic parameters.

Temperature	qe, mg/g	K_L L/mg	R^2
20ºC/ 293K	32.33	1.669	0.995
30ºC/ 303K	23.87	2.330	0.969
40ºC/ 313K	25.20	1.810	0.934
50ºC/ 323K	17.40	4.500	0.955

Figure S4. Adsorption isotherm obtained at different temperatures and used for thermodynamic parameters to be calculated. Process conditions: Ci dye 20-100 mg/L. C ads=1g/L, pH 4.2, under dynamic regime with magnetic mixing speed of 200 rpm

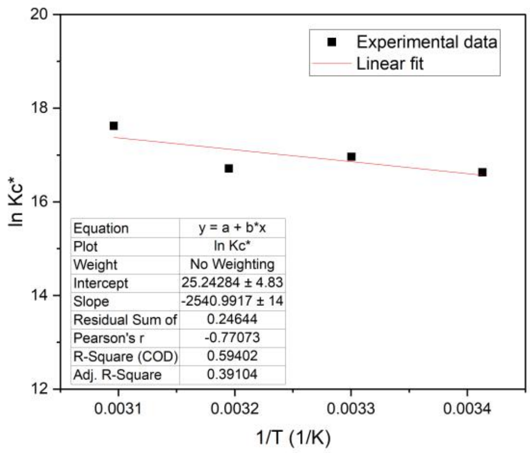

Thermodynamic parameters for reactive yellow adsorption onto various magnetite in aqueous solution

Temp.	ΔG^o, KJ/mol	*ΔH^o, KJ/mol	*ΔS^o, KJ/(mol*K)
20ºC/ 293K	-40.51	21.12	0.206
30ºC/ 303K	-42.73		
40ºC/ 313K	-43.49		
50ºC/ 323K	-47.32		

* With K_L to Kc* unit conversion after (Milonjic 2007)

Figure S5. Van`t Hoff plot of experimental data of reactive yellow adsorption onto plain magnetite and appropriate linear fit, that allowed thermodynamic parameters ΔH^o and ΔS^o to be calculated.

2.5. Magnetite reuse: adsorption desorption studies

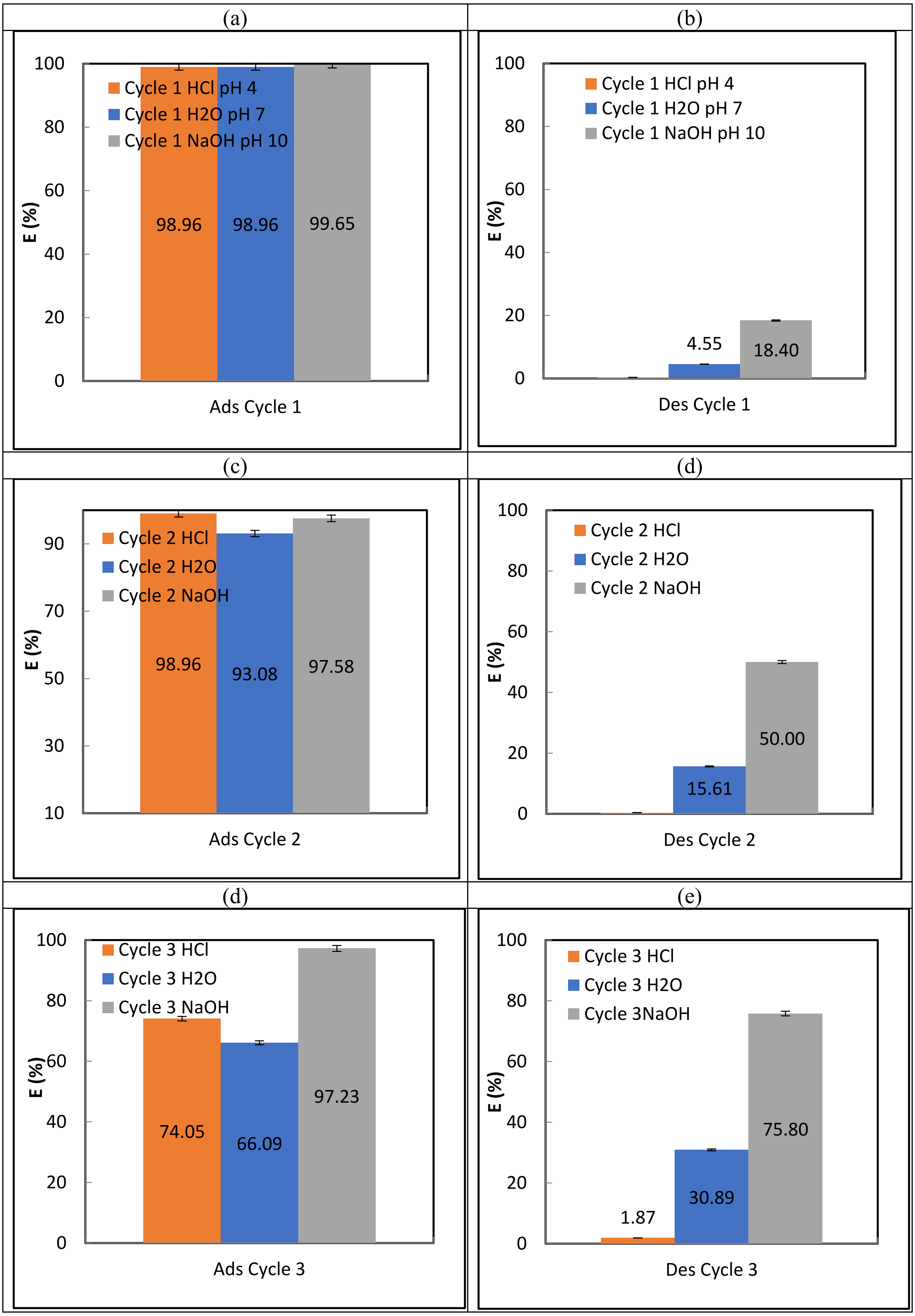

(f)

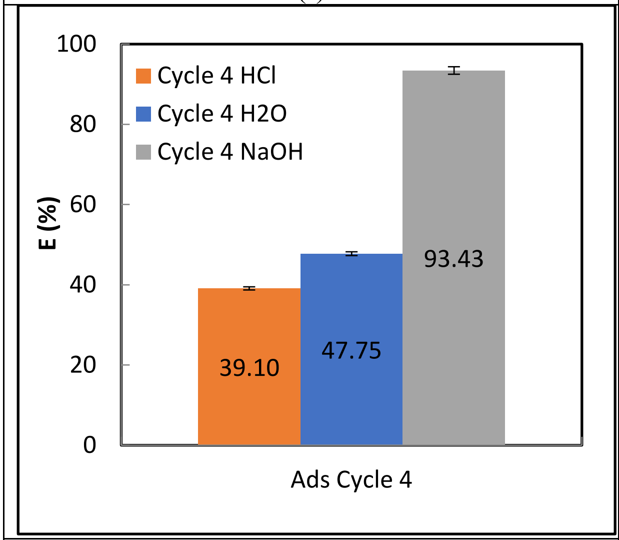

(g)

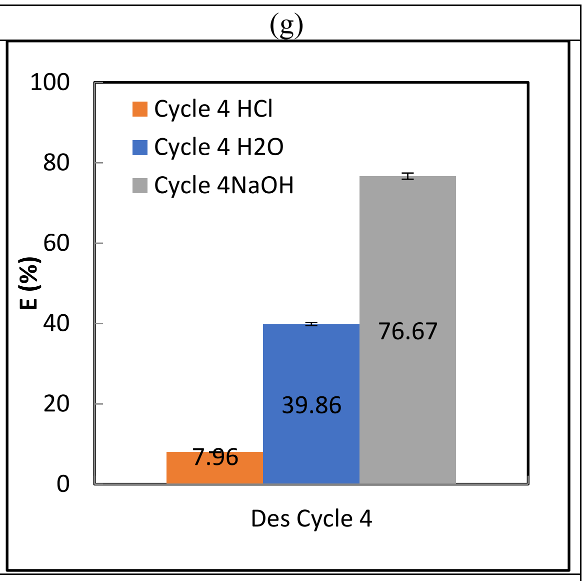

(g)

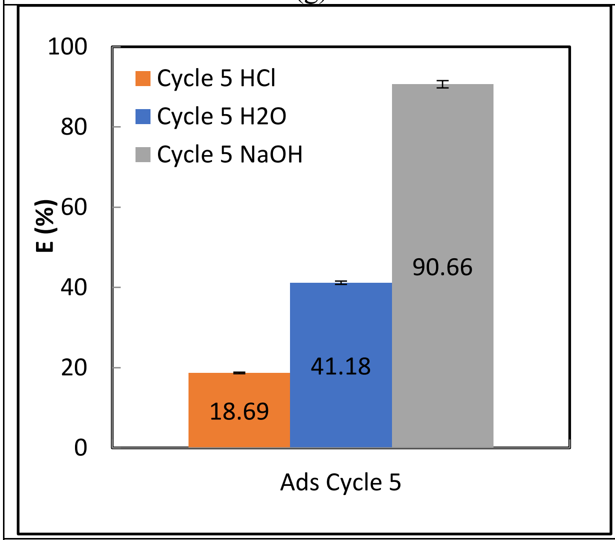

(h)

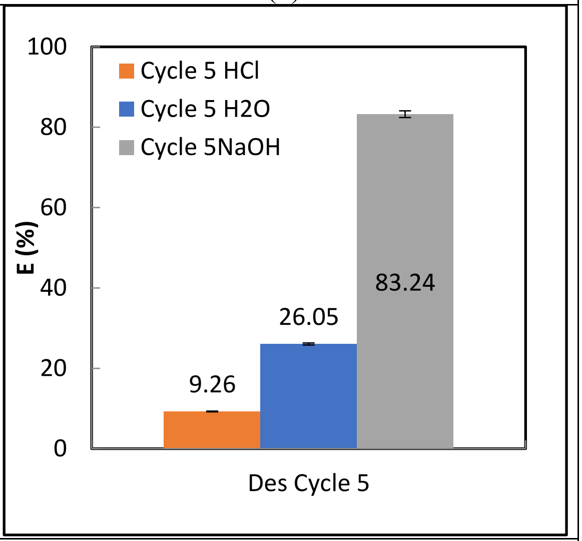

Figure S6. The efficiency of reactive yellow adsorption onto magnetite reused in six consecutive adsorption-desorption cycles, using as eluent between cycles: (a) an acidic solution of HCl 0.1mM of pH 4, (b) a neutral solution of H_2O pH 7 and (c) a basic solution of NaOH 0.1mM of pH 10. Adsorption process conditions: Cdye = 20 mg/L, Cads = 1.0 g/L, pH 4.2, T= 20ºC; Dynamic regime: magnetic stirring at 200 rpm, contact time 60 minutes; Desorption process conditions: C_{eluent} = 0.1mM HCl; 0.1mM NaOH and DDW brought to pH 7 with NaOH 1mM, pH 4, 7 and 10, respectively, C_{ads} = 1.0 g/L, T= 20ºC; Dynamic regime: magnetic stirring at 200 rpm, contact time 10 min

2.6. Comparison of magnetite uptake performance with other materials tested in the literature

Table S2. *Maximum adsorption capacities of different adsorbents tested for dye removal (after Brinza et. al., 2022[14])and Maftei et al 2023[15])*

Materials	Dye	q_{max} (mg/g)	C_i (mg/L)	pH	Refs.
Fe_3O_4 (Prepared in controlled hydrothermal anaerobic conditions, 10-25 nm, rounded plates, SA=84.98 m^2/g,)	Reactive Yellow 84	32.99	10-100	pH 4.2	This study
γ-Fe_2O_3_EG (controlled hydrothermal anaerobic conditions, 50 nm size, spherical size, SA=79.51 m^2/g)	Reactive Yellow 84	39.42	10-100	pH 4	[15]
Bis aldehyde-functionalized silica gel (Prepared as silica gel modified with 2,2′-(hexane-1,6-diylbis(oxy))dibenzaldehyde (SiO_2-NH_2-BH), 40-50nm, spherical lparticles [@])	Reactive Yellow 84	68.02	20	pH 6	[16]
Calcium Carbonate-EDTA 10% (Prepared as calcium carbonate coprecipitation with EDTA -10% Ca-EDTA molar ratio, rhombohedral particles of ca 5 microns size , SA=11.16 m^2/g)	Reactive Yellow 84	39.35	5-60	pH 8	[14]
Hydroxyapatite (Calcium hydroxyapatite HAP was synthesized by double decomposition, solid sieved through 63 µm, SA=137 m^2/g.)	Reactive Yellow 84	50.25	10-40	pH 5	[17]
Cotton fibers^<br><br>Cotton fibers functioned with epichlorohydrin and ammonia water *	Reactive Yellow 84	43.32<br><br>15.90	50-250	pH 3.2	[18]
Maleate-alumoxane nanoparticle [&].SA= 99.83 m^2/g	Reactive Yellow 84	243.9	151.5	pH 4.3	[19]
γ-Fe_2O_3 (co-precipitation method followed by aeration oxidation using ultrasonic vibration, SA=81.61m^2/g ˜)	Congo Red	208.33	20-600	pH 5.9	[20]
γ-Fe_2O_3/chitosan	Methyl Orange	29.41	10-60	pH 2.9	[21]

(commercial maghemite functionalised by solution casting method*)	

[@] Commercial silica gel (60-120 mesh) surface was activated by refluxing with HCl solution (18%) for 24 h, then filtered and repeatedly washed to pH 7. The activated silica was dried in an oven at 150 °C for 24 h. It was functionalised by suspending it in anhydrous toluene and then adding 3-amino propyl trim ethoxy silane (APTES, dissolved in 15 mL dried toluene). The mixture was refluxed for 72 h and the product (SiO_2-NH_2) filtered, washed twice with toluene, twice with ethanol and once with diethyl ether. Finally, it was dried at 70 °C for 24 h before use in adsorption tests.

^The cotton fibres rinsed in H_2SO_4 (2 M) and then in NaOH (2 M). After filtration, they were washed with distilled water and dried at 105 °C; stored in an airtight container at room temperature (25 °C), and soaked for 12 h in distilled water before use.

* aminated cotton fibres (ACFs) preparation: CFs (100 g) were placed into a conical flask with 100 g of an epichlorohydrin solution (ECH) (99.0%). The epichlorohydrin solution in the flask (containing CFs) was then subjected to pH correction (to pH 12) with NaOH solution (1 M), shaked in water bath set at 60 °C, 150 r.p.m. After 24 h, the cotton fibers were drained and washed with distilled water to remove unreacted ECH. After washing, they were put into a solution of ammonia and kept tight for 24 h to ammonizatate. Then, filtered, washed and dried (105 °C). The aminated cotton fibers (ACFs) were soaked for 12 h in distilled water before use.

[&]Carboxylate-alumoxanes preparation: carboxylic acid and boehmite were mixed in deionized water to achieve a homogenous suspension. Maleic acid was obtained by hydrolyze of maleic anhydride with water then ultrasonicate for 20 min, and then refluxed at its boiling point for 10–18.5 h, depending to the type of alumoxane, then filtrated. Both solution-suspensions were mixed carefully to make sure the dissolution of residual-unreacted carboxylic acid. The final resulting suspension was centrifuged and dryed in an oven at 80 °C before use in applications.

˜ A Na_2SO_3 solution (1 M) was added to $FeCl_3$ solution (2 M) dropwise in 1min ultrasonic vibration for a few minutes leading to smaller and more homogenized particles. After turning the colour of solution again, the solution mixed with a NH_3 solution (0.85 M) under ultrasonic vibration. The black precipitate was allowed to crystallize completely for another 30 min, then washed with DDW until the pH was less than 7.5. the pH was adjusted to 3.0 and kept stable for 5 min. The suspension was refluxed under aeration (with air) for 60 min at about 100 °C until the colour of the suspension slowly changed to reddish-brown, washed with DDW by magnetic decantation for several times and dried into powder prior further use.

*commercial magnetic maghemite, ɣ-Fe_2O_3, nanoparticles were dispersed into 2% (w/v) chitosan-dilute acetic acid colloidal solution and ultra-sonicated for 30 min. The viscous mixture solution was cast onto glass plates to form uniform films, and dried at room temperature. The dried films onto glass were immersed into NaOH solution (0.2 M) and flaked away from the glass plates. 2% (v/v) of glutaraldehyde solution was added dropwise with continuous stirring for 30 min in a water bath at 60 °C, to improve the acid resistance of the films, and finally washed with alcohol, N, N-dimethylformamide and double distilled water for two times. It was dried at 60 °C till constant weight, before further use in adsorption laboratory tests.

2.7. Physical and chemical characteristics of the Blue Lake water used in adsorption studies.

Table S3. Selected physical and chemical characteristics of the Blue Lake water used in adsorption studies.

Parameters	SI Unit	Value

Temperature	°C	20
pH	-	4.1
Conductivity	µS/cm la 20°C	151
Total dissolved solids	mg/L(ppm)	76
Na (588)*	mg/L	4.381
K (766)*	mg/L	6.790
Mg (285)*	mg/L	0.970
Fe (248)*	mg/L	0.000
Co (240)*	mg/L	0.013
Cu (324)*	mg/L	0.008
Zn (213)*	mg/L	0.738
RY 84	mg/L	10

* Measured by ContrAA 800D FAAS

References

1. Qiu, H., et al., *Critical review in adsorption kinetic models.* Journal of Zhejiang University-SCIENCE A, 2009. **10**(5): p. 716-724.
2. Wang, J. and X. Guo, *Adsorption kinetic models: Physical meanings, applications, and solving methods.* Journal of Hazardous Materials, 2020. **390**: p. 122156.
3. Ho, Y.-S., *Review of second-order models for adsorption systems.* Journal of Hazardous Materials, 2006. **136**(3): p. 681-689.
4. Ho, Y.S. and G. McKay, *A Comparison of Chemisorption Kinetic Models Applied to Pollutant Removal on Various Sorbents.* Process Safety and Environmental Protection, 1998. **76**(4): p. 332-340.
5. Armagan, B., M. Turan, and D. Karadag, *Adsorption of Different Reactive Dyes onto Surfactant-Modified Zeolite: Kinetic and Equilibrium Modeling*, in *Survival and Sustainability: Environmental concerns in the 21st Century*, H. Gökçekus, U. Türker, and J.W. LaMoreaux, Editors. 2011, Springer Berlin Heidelberg: Berlin, Heidelberg. p. 1237-1254.
6. Fila, D., Z. Hubicki, and D. Kołodyńska, *Applicability of new sustainable and efficient alginate-based composites for critical raw materials recovery: General composites fabrication optimization and adsorption performance evaluation.* Chemical Engineering Journal, 2022. **446**: p. 137245.
7. Elovich, S.Y. and O.G. Larinov, *Theory of adsorption from solutions of non electrolytes on solid (I) equation adsorption from solutions and the analysis of its simplest form, (II) verification of the equation of adsorption isotherm from solutions.* Izv. Akad. Nauk. SSSR, Otd. Khim. Nauk, 1962. **2**(2): p. 209-216.
8. McLintock, I.S., *The Elovich Equation in Chemisorption Kinetics.* Nature, 1967. **216**(5121): p. 1204-1205.
9. Lagergren, S., *Zur theorie der sogenannten adsorption gelöster stoffe, Kungliga Svenska Vetenskapsakademiens. Handlingar*, 1886. **24**(4): p. 1-39.
10. Simonin, J.-P., *On the comparison of pseudo-first order and pseudo-second order rate laws in the modeling of adsorption kinetics.* Chemical Engineering Journal, 2016. **300**: p. 254-263.
11. Weber, W.J. and J.C. Morris, *Kinetics of adsorption on carbon from solution.* ASCE Sanit. Eng. Div. J., 1963. **1**(2): p. 1-2.
12. Al-Ghouti, M.A. and D.A. Da'ana, *Guidelines for the use and interpretation of adsorption isotherm models: A review.* Journal of Hazardous Materials, 2020. **393**: p. 22.
13. Wang, J. and X. Guo, *Adsorption isotherm models: Classification, physical meaning, application and solving method.* Chemosphere, 2020. **258**: p. 127279.

14. Brinza, L., et al., *Advanced removal of Reactive Yellow 84 azo dye using functionalised amorphous calcium carbonates as adsorbent.* Scientific Reports, 2022. **12**(1).
15. Maftei, A.E., et al., *Nanocrystalline structured ethylene glycol doped maghemite for persistent pollutants removal.* Environmental Science: Water Research and Technology, 2023. **9**: p. 1634-1645.
16. Banaei, A., et al., *Synthesis of silica gel modified with 2,2′-(hexane-1,6-diylbis(oxy)) dibenzaldehyde as a new adsorbent for the removal of Reactive Yellow 84 and Reactive Blue 19 dyes from aqueous solutions: Equilibrium and thermodynamic studies.* Powder Technology, 2017. **319**: p. 60-70.
17. Barka, N., et al., *Removal of Reactive Yellow 84 from aqueous solutions by adsorption onto hydroxyapatite.* Journal of Saudi Chemical Society, 2011. **15**(3): p. 263-267.
18. Jóźwiak, T., et al., *The use of aminated cotton fibers as an unconventional sorbent to remove anionic dyes from aqueous solutions.* Cellulose, 2020. **27**(7): p. 3957-3969.
19. Ghabaee, S., et al., *Synthesis and characterization maleate-alumoxane nanoparticles for removal of reactive yellow 84 dye from aqueous solution.* Advanced Powder Technology, 2020. **31**(5): p. 2061-2071.
20. Afkhami, A. and R. Moosavi, *Adsorptive removal of Congo red, a carcinogenic textile dye, from aqueous solutions by maghemite nanoparticles.* J Hazard Mater, 2010. **174**(1-3): p. 398-403.
21. Jiang, R., et al., *Removal of methyl orange from aqueous solutions by magnetic maghemite/chitosan nanocomposite films: Adsorption kinetics and equilibrium.* Journal of Applied Polymer Science, 2012. **125**(S2): p. E540-E549.
